# A quantitative physical model of the TMS-induced discharge artifacts in EEG

**DOI:** 10.1371/journal.pcbi.1006177

**Published:** 2018-07-17

**Authors:** Dominik Freche, Jodie Naim-Feil, Avi Peled, Nava Levit-Binnun, Elisha Moses

**Affiliations:** 1 Sagol Center of Brain and Mind, Ivcher School of Psychology, Interdisciplinary Center (IDC), Herzliya, Israel; 2 Department of Neurobiology, Weizmann Institute of Science, Rehovot, Israel; 3 Department of Physics of Complex Systems, Weizmann Institute of Science, Rehovot, Israel; 4 Rappaport Faculty of Medicine, Technion, Haifa, Israel; 5 Institute for Psychiatric Studies, Sha’ar Menashe Mental Health Center, Sha’ar Menashe, Israel; Ghent University, BELGIUM

## Abstract

The combination of Transcranial Magnetic Stimulation (TMS) with Electroencephalography (EEG) exposes the brain’s global response to localized and abrupt stimulations. However, large electric artifacts are induced in the EEG by the TMS, obscuring crucial stages of the brain’s response. Artifact removal is commonly performed by data processing techniques. However, an experimentally verified physical model for the origin and structure of the TMS-induced discharge artifacts, by which these methods can be justified or evaluated, is still lacking. We re-examine the known contribution of the skin in creating the artifacts, and outline a detailed model for the relaxation of the charge accumulated at the electrode-gel-skin interface due to the TMS pulse. We then experimentally validate implications set forth by the model. We find that the artifacts decay like a power law in time rather than the commonly assumed exponential. In fact, the skin creates a power-law decay of order 1 at each electrode, which is turned into a power law of order 2 by the reference electrode. We suggest an artifact removal method based on the model which can be applied from times after the pulse as short as 2 milliseconds onwards to expose the full EEG from the brain. The method can separate the capacitive discharge artifacts from those resulting from cranial muscle activation, demonstrating that the capacitive effect dominates at short times. Overall, our insight into the physical process allows us to accurately access TMS-evoked EEG responses that directly follow the TMS pulse, possibly opening new opportunities in TMS-EEG research.

## Author summary

The combined use of transcranial magnetic stimulation (TMS) with electroencephalography (EEG) is rapidly becoming a useful diagnostic and research tool for measuring the brain’s global response to localized and abrupt stimulations. However, large electric artifacts that are induced in the EEG by the TMS must be removed from the EEG signal, since they are unrelated to brain activity and obscure crucial stages of the brain’s response. Here we show that a simple physical model of skin impedance explains the observed artifact decay, and enables the efficient removal of the artifact signal in an automatable manner. We thus discern the EEG response precisely from about 2 milliseconds after the TMS pulse, showing that the artifact is dominated by capacitive rather than muscular effects.

## Introduction

The combined use of transcranial magnetic stimulation (TMS) with electroencephalography (EEG) has become a well-established method in neuroscience. It is used for functional cortical mapping [1] and has been suggested for the identification of biomarkers [2, 3]. However, the concomitant application of TMS-EEG remains a challenge [4, 5], mainly due to the effect of the electromagnetic pulse of TMS on the EEG. Although technical advances have led to improved amplifiers that allow continuous recording during pulse application without amplifier saturation, the TMS pulse leads to large artifacts in the EEG recording. These artifacts are orders of magnitude larger than the physiological brain activity, and persist from a few to hundreds of milliseconds [5]. This usually makes it impossible to directly obtain physiological information from the artefactual EEG without further experimental precautions or additional algorithmic treatment. Despite ongoing efforts of the past two decades to address the artifact problem, both the origin and the structure of the artifact are still under debate. Likewise, no generally accepted algorithm is available that can remove the artifacts without unintentionally and significantly altering physiological information contained in the EEG recordings. In this paper, we address these issues.

Depending on the TMS device, the magnetic pulse lasts for around 300 μs and changes in time, which in turn generates a spatially and temporally varying electric field of the same duration. Both physiological and technical artifacts are associated with these fields (for a comprehensive review see [3] and [5]). Specifically, the induction current can charge various capacitances along its path. This applies to the double-layer capacitance of the electrode-gel interface and to the gel-skin interface [6] as well as to capacitances of both the upper and the deeper skin layers [7, 8]. In this paper, we refer to the entirety of all artifact components which are a consequence of the TMS application as the TMS-induced artifacts. To refer to artifact components of a specific origin, we use the following terminology: We call the artifact due to current induction during the TMS pulse the pulse artifact. Due to its sharp peak-like shape, we also denote it the ‘initial peak’. Artifacts resulting from relaxation of any capacitances that were charged by current flow during the TMS pulse are referred to as discharge artifacts. Artifacts resulting from muscle, auditory, and somatosensory activation, are called muscle, auditory and somatosensory artifacts, respectively.

Different methods to deal with these TMS-induced artifacts have been suggested, both by experimental adaptations [9, 10] and by post-processing of the data [11–17, 17–20]. Out of the post-processing group, only [19, 20] are based on a physical assumption of exponential capacitive discharge as origin of the decaying artifacts. In [21], the artifact is modeled by the sum of two exponentials. The other methods postulate the existence of specific properties of artifacts in EEG signals and suggest methods from statistical signal processing to remove the artifacts [3]. However, the lack of a detailed and experimentally validated physical model has profound consequences: It hampers a deeper understanding of the various methods, and an assessment of their advantages and disadvantages. It also impedes an evaluation of the validity of methods that attempt to separate the artifacts from the brain activity. Perhaps the most important issue with the current methods, however, is that the validity of the assumptions on which they are based is not known [3, 22]. There is therefore an urgent need for a physical model.

To progress towards a detailed physical artifact model, we go beyond the standard, ‘lumped’ model that describes the skin as a leaky capacitance, to one that introduces the spatial extent of the skin, realizing that the gel-skin interface and the scalp are both loci of dominant and large capacitances [10]. These capacitors will typically be charged via induction of current in the wires and electrodes [9]. Indeed, a detailed examination of the skin structure shows that interfaces within the skin are natural electrolytic capacitors. The skin consists of three principal layers, the outermost being the epidermis, followed by the dermis, and then the subcutaneous tissue. It is the epidermis that plays the major role in the electrode-skin connection [6, 23], new cells grow at its innermost side and upon maturation organize into different layers of the epidermis. In the last maturation stage of the epidermis, the cells form the stratum corneum layer, with a thickness of ten to a few tens of micrometers. The stratum corneum consists of dead, keratinized cells embedded in extracellular space that is filled with lipids [23], while the extracellular space of other layers of the epidermis and skin is aqueous. Therefore, the stratum corneum acts as a hydrophobic dielectric separating two aqueous electrolytes, namely the gel above and the remaining skin below [24]. We refer to this structure as the skin capacitor.

The lower layers also have capacitance [7, 8], but predominantly act as volume conductors. However, there are small and localized impurities, which provide diffusion routes across the stratum corneum. These are constituted by skin appendages such as sweat glands and hair follicles. The hair follicles are connected to sebaceous glands, which fill the space around them with hydrophobic grease. This effectively turns the gel-skin interface into a two-dimensional array of capacitors with occasional small leaks that function as resistors.

The scheme of this paper is as follows. In the Methods section, we introduce the model for the voltage relaxation dynamics and discuss some of its experimentally testable implications, most prominently the mathematical result that the tails of the voltage decay follow a specific power law. The manner of testing is also described in the Methods. The Results section lays out the tests, which first include the measurement of TMS-EEG from human knee and from phantom heads (melons). Then the voltage decay from TMS stimulation that is prefaced by skin preparation is examined. We also look systematically into a variety of effects such as the difference between passive versus active EEG systems and the effects of varying the sampling rate. Finally, we demonstrate on real data how our method can be used to remove the discharge artifacts from various EEG recordings. This shows that discharge artifacts dominate over muscle artifacts at short times.

## Materials and methods

### A quantitative physical model for the TMS-EEG artifact

**Fig 1:**
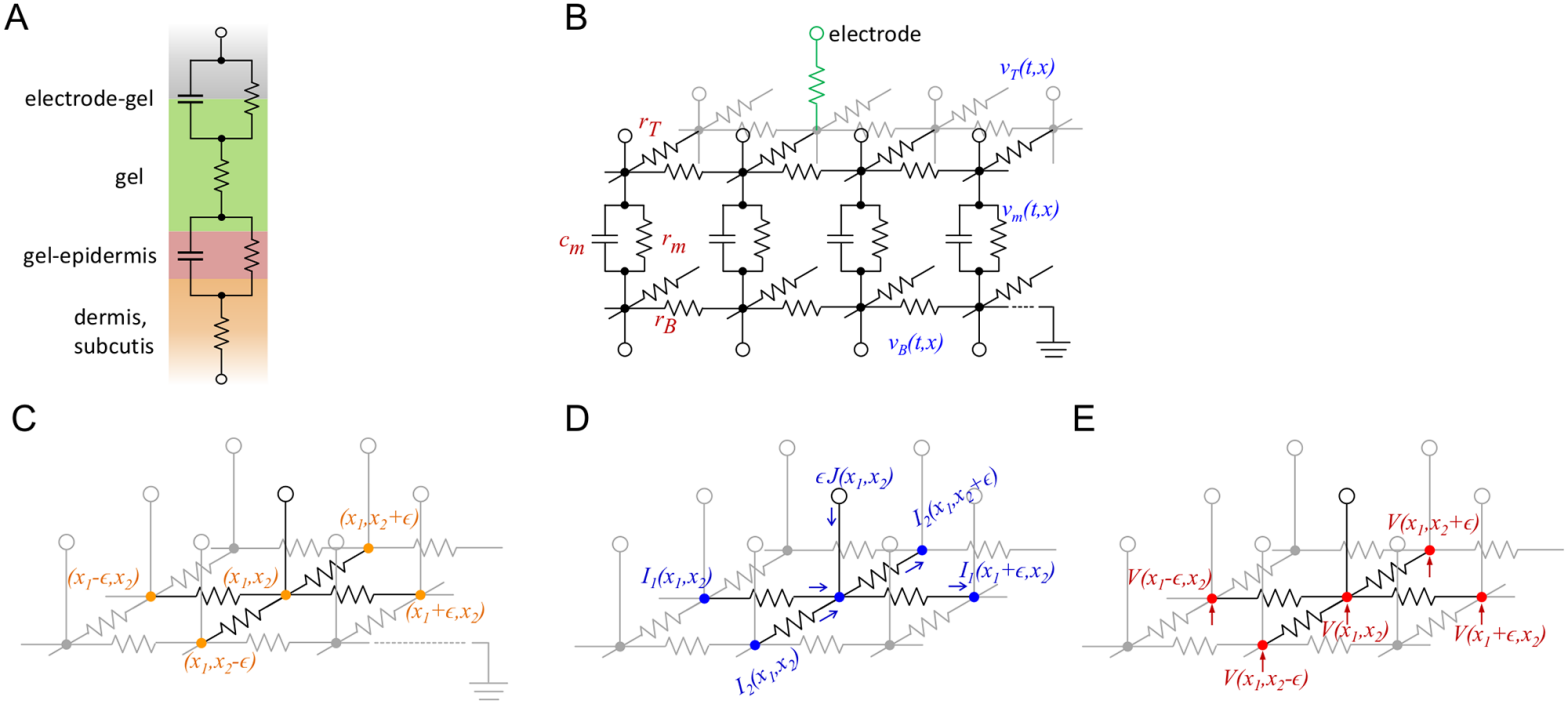
Equivalent circuits for the electrode-gel-skin interface. (A) Description of the electrode-skin-gel interface as lumped element model. The resistance and capacitance of the skin incorporate spatially varying properties. (B) Distributed circuit model for the spatial extent of the dermal interface. (C) The topology of a single-layer infinite regular grid with edge length *ϵ*. A node *x* = (*x*_1_, *x*_2_) has four direct neighbors (*x*_1_±*ϵ*, *x*_2_), (*x*_1_, *x*_2_±*ϵ*). (D) The currents in direction *x*_1_ and *x*_2_ are denoted by *I*_1_ and *I*_2_, respectively. Note that the injected current *J* is required to scale with *ϵ*. (E) The voltages at the nodes are denoted by *V*.

Our physical model looks how charges that accumulate on the electrode-gel-skin interface undergo capacitive discharge, which is manifested in the slowly decaying artifacts measured in the EEG. The standard textbook model of the electrode-gel-skin interface [6] lumps the combined capacitive effects into two leaky capacitors in series (Fig 1A), giving exponential discharge curves. In contrast, we specifically introduce the lateral extent of the interface in the model, i.e. we consider that initially the main motion of the charges is transverse on the surface of the scalp at the electrode. We still describe the motion of the charges in the third dimension, i.e. into the skin, according to the simplistic model of Fig 1A.

We treat the gel on the scalp and the deep layers of the skin as purely resistive, while the gel-epidermis interface is both resistive and capacitive. The structure is thus an extended capacitor-resistor network in which the charges have to diffuse in the conductor on top before they can cross the dielectric layer to the layers below. This has profound consequences on the discharge dynamics, yielding different temporal regimes that can be linked to details of the TMS artifacts. Another crucial component of the model is to include the interplay of the discharge dynamics at a recording electrode with the discharge dynamics in the reference electrode.

To derive the model equations, we next apply a well-known formal homogenization technique to the resistor-capacitor network. This formalism is a standard one, used in the derivation of the cable equation in one dimension, as presented in textbooks such as [25]. Cable equations are models for the voltage difference across two resistive domains connected by resistive-capacitive junctions. Our two dimensional derivation is more general in that it obtains equations for the voltage distributions on each of the domains. In particular, the standard two-dimensional cable equation can be obtained from our model as a special case.

#### The voltage distribution on a resistive-capacitive sheet

We consider an infinite two-dimensional regular grid of resistors. It consists of nodes (*x*_1_, *x*_2_), where *x*_1_ and *x*_2_ are all integer multiples of the edge length *ϵ*. Every edge is a resistor with resistance *ϵr*. A current *J*(*x*_1_, *x*_2_) is assumed to be injected at every node from a source that is external to the sheet. The voltage at each node of the grid is denoted by *V* and the currents through the edges in *x*_1_-direction by *I*_1_ and in *x*_2_-direction by *I*_2_. We assume that the grid is grounded at infinity. Both currents *I*_1_ and *I*_2_ are defined to flow in the direction of positive *x*_1_ and *x*_2_. The model is summarized in Fig 1C–1E and can be viewed as a two-dimensional version of the discrete transmission line model. By Kirchhoff’s current law, a relation between the currents at every node can be derived,

$$\begin{array}{c} I_{1}\left(x_{1},x_{2}\right)+I_{2}\left(x_{1},x_{2}\right)+\epsilon J\left(x_{1},x_{2}\right)=I_{1}\left(x_{1}+\epsilon ,x_{2}\right)+I_{2}\left(x_{1},x_{2}+\epsilon \right)\;, \end{array}$$

and by Ohm’s law, the voltage difference over an edge in direction *x*_1_ and *x*_2_, respectively, is

$$\begin{array}{c} V\left(x_{1},x_{2}\right)-V\left(x_{1}+\epsilon ,x_{2}\right)=\epsilon rI_{1}\left(x_{1}+\epsilon ,x_{2}\right)\;,\\ V\left(x_{1},x_{2}\right)-V\left(x_{1},x_{2}+\epsilon \right)=\epsilon rI_{2}\left(x_{1},x_{2}+\epsilon \right)\;. \end{array}$$

By rearranging and formally replacing the finite differences by derivatives (corresponding to formally taking the limit *ϵ* → 0), we obtain

$$\begin{array}{cc} \partial_{x_{1}}I_{1}\left(x_{1},x_{2}\right)+\partial_{x_{2}}I_{2}\left(x_{1},x_{2}\right) & =-J(x_{1},x_{2})\;,\\ \partial_{x_{1}}V\left(x_{1},x_{2}\right) & =rI_{1}\left(x_{1},x_{2}\right)\;,\\ \partial_{x_{2}}V\left(x_{1},x_{2}\right) & =rI_{2}\left(x_{1},x_{2}\right)\;, \end{array}$$

where $\left(x_{1},x_{2}\right)\in R^{2}$. Taking the derivative another time, we obtain

$$\begin{array}{c} \partial_{x_{1}x_{1}}^{2}V\left(x_{1},x_{2}\right)=r\partial_{x_{1}}I_{1}\left(x_{1},x_{2}\right)\;,\\ \partial_{x_{2}x_{2}}^{2}V\left(x_{1},x_{2}\right)=r\partial_{x_{2}}I_{2}\left(x_{1},x_{2}\right)\;, \end{array}$$

which can be summarized as

$$\begin{array}{c} \Delta V\left(x_{1},x_{2}\right)=-rJ\left(x_{1},x_{2}\right)\;, \end{array}$$

and the condition of grounding at infinity becomes

$$\begin{array}{c} V\left(x_{1},x_{2}\right)\to 0\;\text{for}\;|x_{1}{|}^{2}+{\left|x_{2}\right|}^{2}\to \infty \;. \end{array}$$

This is a Poisson equation in the plane $R^{2}$.

Next, we consider the resistive-capacitive double grid as shown in Fig 1B, which is the basis of our model. It consists of two infinite regular grids that are purely resistive, with one located above the other such that they can be designated top and bottom layer. Each node of the top layer is connected to the bottom layer by a leaky capacitor. We assume that all leaky capacitors have resistance *r*_*m*_ and capacitance *c*_*m*_. The respective edge resistivities of the top and bottom layer are denoted by *r*_*T*_ and *r*_*B*_, and the corresponding top and bottom voltages by *v*_*T*_ and *v*_*B*_. Note that *v*_*T*_ and *v*_*B*_ now also depend on time. We require both layers to be grounded at infinity. By slight abuse of notation, we denote a top node as well as its corresponding bottom node by the same symbol $x=\left(x_{1},x_{2}\right)\in R^{2}$, because there is no risk of confusion when they are used as argument of quantities related to either the top or bottom grid, e.g. *v*_*T*_(*t*, *x*) and *v*_*B*_(*t*, *x*).

The current *J* from a top to a bottom node is the sum of a capacitive part and resistive part,

$$\begin{array}{c} J(x)=c_{m}\partial_{t}(v_{T}\left(x\right)-v_{B}\left(x\right))+r_{m}(v_{T}\left(x\right)-v_{B}\left(x\right))\;. \end{array}$$

Using the same formal homogenization as above, we obtain the following system of equations for *v*_*T*_ and *v*_*B*_, which can be written in matrix form,

$$\begin{array}{c} c_{m}[\begin{array}{cc} 1 & -1\\ -1 & 1 \end{array}]\partial_{t}[\begin{array}{c} v_{T}\\ v_{B} \end{array}]=[\begin{array}{cc} 1/r_{T} & 0\\ 0 & 1/r_{B} \end{array}]\Delta [\begin{array}{c} v_{T}\\ v_{B} \end{array}]-\frac{1}{r_{m}}[\begin{array}{cc} 1 & -1\\ -1 & 1 \end{array}][\begin{array}{c} v_{T}\\ v_{B} \end{array}]\;. \end{array}$$ (1)

A single equation for the cross-layer voltage difference *v* = *v*_*T*_ − *v*_*B*_ can be obtained from (1) by subtraction. This is the two-dimensional version of the cable equation,

$$\begin{array}{c} \tau \partial_{t}v=\lambda^{2}\Delta v-v \end{array}$$ (2)

with a length constant *λ* and a time constant *τ*, which are expressed in the form *λ*^2^ = *r*_*m*_/(*r*_*T*_+ *r*_*B*_) and *τ* = *r*_*m*_ *c*_*m*_. Using (2), the voltage response of the cross-layer to a localized initial voltage impulse *δ*_*t* = 0, *x* = (0, 0)_ can be obtained. Its spatial and temporal dynamics yield as a solution the two-dimensional density response *g*(*t*, *x*) with

$$\begin{array}{c} g\left(t,x\right)=\frac{\tau }{4\pi \lambda^{2}t}\exp(-\frac{x^{2}\tau }{4\lambda^{2}t}-\frac{t}{\tau })\;. \end{array}$$ (3)

Using *g*(*t*, *x*), we can derive a solution to (1). To this end, we decouple the equations using the eigenvalue decomposition

$$\begin{array}{c} {}[\begin{array}{cc} r_{T} & -r_{T}\\ -r_{B} & r_{B} \end{array}]=WDW^{-1} \end{array}$$

where

$$\begin{array}{c} W=[\begin{array}{cc} -r_{T}/r_{B} & 1\\ 1 & 1 \end{array}],\;W^{-1}=\frac{1}{r_{B}+r_{T}}[\begin{array}{cc} -r_{B} & r_{B}\\ r_{B} & r_{T} \end{array}],\;D=[\begin{array}{cc} r_{T}+r_{B} & 0\\ 0 & 0 \end{array}] \end{array}$$

by using the transformation

$$\begin{array}{c} {}[\begin{array}{c} {\tilde{v}}_{T}\\ {\tilde{v}}_{B} \end{array}]=W^{-1}[\begin{array}{c} v_{T}\\ v_{B} \end{array}]\;, \end{array}$$

such that we can write (1) as

$$\begin{array}{c} \tau \partial_{t}{\tilde{v}}_{T}=\lambda^{2}\Delta {\tilde{v}}_{T}-{\tilde{v}}_{T}\;, \end{array}$$ (4)

$$\begin{array}{c} 0=\Delta {\tilde{v}}_{B}\;, \end{array}$$ (5)

Solving (5) with ${\tilde{v}}_{B}=0$ enforces the initial condition for (4),

$$\begin{array}{c} {\tilde{v}}_{T}\left(0,x\right)=v_{B}\left(0,x\right)\;. \end{array}$$ (6)

Because (4) is a two-dimensional cable equation, it can be solved in terms of *g*(*t*, *x*). By transforming back, it can be seen that condition (6) is satisfied when a given localized voltage difference *v*_0_(*x*) is prescribed at time *t* = 0 between the node (0, 0) of the top and bottom layers, i.e., for the initial conditions for (1)

$$\begin{array}{c} v_{T}\left(0,x\right)=\frac{r_{T}}{r_{T}+r_{B}}v_{0}\delta_{t=0,x=(0,0)}\;,\\ v_{B}\left(0,x\right)=\frac{-r_{B}}{r_{T}+r_{B}}v_{0}\delta_{t=0,x=(0,0)}\;. \end{array}$$

Then the solution to (1) has the form

$$\begin{array}{c} v_{T}\left(t,x\right)=\frac{v_{0}r_{T}}{r_{T}+r_{B}}g\left(t,x\right)\;,\\ v_{B}\left(t,x\right)=\frac{-v_{0}r_{B}}{r_{T}+r_{B}}g\left(t,x\right)\;. \end{array}$$

In other words, the surface voltages on the top and bottom layer in response to a localized voltage pulse are proportional to the voltage difference between the layers.

#### Identification of three discharge regimes and the emergence of power law decays

We can identify three time regimes in the behavior of *g* for fixed *x* as defined in (3). At very short times *t* ≪ *x*^2^ *τ*/*λ*^2^, the exponential in *g*(*t*, *x*) is close to zero. Then, at an intermediate time scale *x*^2^ *τ*/*λ*^2^ ≪ *t* ≪ *τ*, the exponential approaches a value of 1. In this intermediate regime the behavior of *g* exhibits a 1/*t* power law dynamics. Finally, at very long times *t* ≫ *τ*, the decay dynamics are exponential. This corresponds to the intuitive understanding that the diffusion process within the layer can arise when charge density equilibration in the lateral direction (controlled by *r*_*B*_ and *r*_*T*_) meets less resistance than in the transversal direction (controlled by *r*_*m*_).

Up to now we discussed *g*, and we can extend the investigation to the voltages *v*_*T*_ and *v*_*B*_. For any arbitrary initial distribution of voltage differences between the layers *v*_0_(*x*), by convolution with *g* we obtain the general solution to (2), *v* = *v*_0_*g. If *v*_0_ is reasonably well behaved then *v* will also be characterized by these three regimes. This also means that the physically relevant distributions *v*_*T*_ and *v*_*B*_ will similarly display these temporal regimes, and in particular the 1/*t* power law decay.

#### Voltage difference between electrodes is a differentiator

EEG usually requires referencing the voltage at each electrode to a particular electrode, and therefore the process of taking the voltage difference between electrodes must be included in our model. This effectively acts as a differentiator. Diffusion leads to a 1/*t* dynamics, as described above. The differentiation, which occurs when subtracting the initial voltage distribution at different electrodes that differ slightly from each other in their initial width, changes the 1/*t* power law regime to a 1/*t*^2^ regime.

To explain how the differentiation comes about, we consider the initial voltage distribution at different electrodes. These are chosen for convenience to be Gaussian, and may vary in their initial width for a number of physical reasons. For convenience we consider two electrodes and prescribe initial conditions on them in the form of Gaussian densities, which can be either all positive or all negative. Such densities are described in terms of the impulse *g*(*σ*, *x*) with the time parameter *σ* leading to a spatial spread *λ*^2^ *σ*/*τ*. The initial Gaussian at the first electrode is denoted *v*_1_(0, *x*) = *g*(*σ*_1_, *x*_1_ − *x*) and the other by a slightly different *v*_2_(0, *x*) = *g*(*σ*_2_, *x*_2_ − *x*) where *x*_1_, *x*_2_ indicate the locations of the electrodes, and *σ*_1_, *σ*_2_ specify slightly different variances in the initial Gaussians. To subtract the voltages on these two electrodes we expand *g* and obtain the voltage difference

$$\begin{array}{cc} & v_{1}\left(t,x_{1}\right)-v_{2}\left(t,x_{2}\right)=g\left(\sigma_{1}+t,0\right)-g\left(\sigma_{2}+t,0\right)\\ & =\left(\sigma_{2}-\sigma_{1}\right)\partial_{t}g\left(\sigma_{1}+t,0\right)+O\left({\left(\sigma_{2}-\sigma_{1}\right)}^{2}\right)\\ & \approx \left(\sigma_{2}-\sigma_{1}\right)/t^{2} \end{array}$$

where we exploit the translational invariance of (2) and its linear dependence on the initial conditions to evaluate the difference of the initial conditions at *x* = (0, 0). From the final form we obtain for the voltage difference we conclude that after reference subtraction the voltage at an electrode behaves like 1/*t*^2^.

#### Accommodating electrode geometries and charge distributions

**Fig 2:**
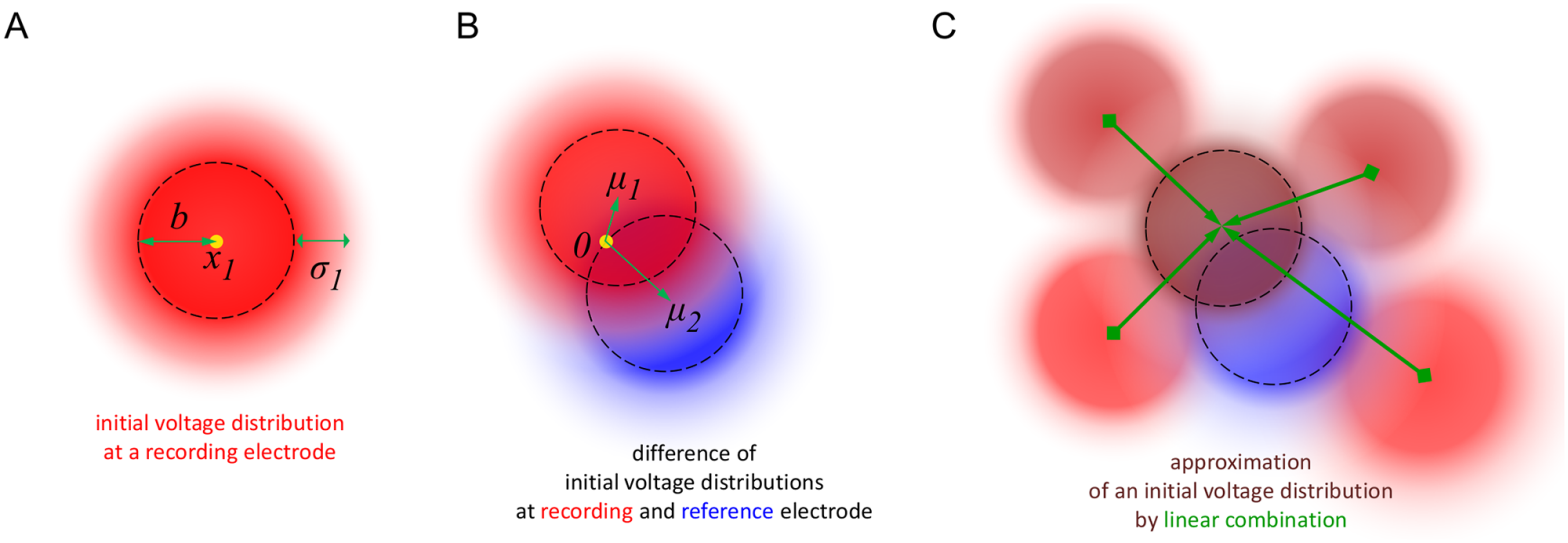
The initial voltage distribution in the skin capacitor after the pulse as described by the model. (A) The initial voltage distribution (shaded red) underneath a recording electrode located at *x*_1_ modelled as a box function *B* with radius *b* (dashed) convolved with a Gaussian with variance *σ*_1_. Darker color means higher density, white means 0. More intense color corresponds to larger voltage amplitude. (B) The initial voltage distribution can have a displacement *μ*_1_ w.r.t. its center *x*_1_. The reference electrode (shaded blue) also has parameters *σ*_2_ and *μ*_2_. (C) Some initial voltage distributions can be selected such that their linear combination (green) may approximate the initial voltage distributions at all remaining electrodes.

The model we presented above is not able reproduce the complete variations of artifact shapes which can be measured at different electrodes. Indeed, two important properties of the electrode-gel-skin contact have to be taken into account. The first is the actual physical structure of the electrode. The surface geometry of an electrode is modelled by convolution of the voltage at the electrode with a spatial ‘box function’ *B*. In a simple scenario we define *B* to be equal to 1/*πb*^2^ within a radius of size *b* (which may depend on the particular electrode), and 0 elsewhere. The voltage measured at the electrode is then the difference of two Gaussians convolved with this box function (see Fig 2A). The evaluation of *v*_1_(*t*, *x*_1_) − *v*_2_(*t*, *x*_2_) then amounts to the computation

$$\begin{array}{cc} v_{1}\left(t,x_{1}\right)-v_{2}\left(t,x_{2}\right) & =[B*g(\sigma_{1}+t,x-x_{1})]\left(x_{1}\right)-[B*g(\sigma_{2}+t,x-x_{2})]\left(x_{2}\right)\\ & =[B*(g\left(\sigma_{1}+t,x\right)-g\left(\sigma_{2}+t,x\right))]\left(0\right)\;. \end{array}$$

The second property concerns small deviations of the Gaussian-shape initial voltage distributions at different electrodes. In fact, they can differ not only in variance but also in their locations with respect to the electrodes. This corresponds to *v*_1_ with *v*_1_(0, *x*) = *g*(*σ*_1_, *x*_1_+ *μ*_1_ − *x*) where *μ*_1_ denotes a (small) displacement vector relative to *x*_1_ (see Fig 2B). Also *v*_2_ can be defined in this way with parameters *σ*_2_ and *μ*_2_. The generalized formula for *v*_1_(*t*, *x*_1_) − *v*_2_(*t*, *x*_2_) is computed below, and behaves like 1/*t*^2^ to leading order. The width of the electrode is specified by *b*, the initial width of the Gaussian distribution of the potential by *σ*, and the offset of this Gaussian from the center of the electrode by *μ*. We thus have the three parameters, *σ* in units of *τ*, *μ* and *b* in units of *λ*. In practice, *b* is used to fine-tune the form of the artifact, and if *b* = 0 then the results do not change much. In this case the function *B*(*x*) = *δ*_*x* = (0, 0)_ can be used as the box function.

It remains to verify that these two mode generalizations do not change the power law regime. To show this we consider two electrodes *i* = 1, 2. The location of electrode *i* is *x*_*i*_. Its initial charge distribution is Gaussian (characterized by a variance *σ*_*i*_) convolved with a spatial box function *B* (characterized by a radius *b*). The vector *μ*_*i*_ specifies a small displacement relative to the center *x*_*i*_ of the electrode location. Therefore, we define the cross-layer voltage at electrode *i* as an average over the spatial extent of the electrode,

$$\begin{array}{cc} v_{i}\left(t,x_{i}\right) & =(B*g(t+\sigma_{i},\mu_{i}+x-x_{i}))\left(x_{i}\right)\\ & =\frac{1}{\pi b^{2}}\int_{|y|\leq b}g\left(t+\sigma_{i},\mu_{i}-y\right)dy \end{array}$$

and we can hence write the voltage difference *v*_1_(*t*, *x*_1_) − *v*_2_(*t*, *x*_2_) of electrode 1 and 2 as

$$\begin{array}{cc} \frac{1}{\pi b^{2}} & \int_{|y|\leq b}g\left(t+\sigma_{1},\mu_{1}-y\right)-g\left(t+\sigma_{2},\mu_{2}-y\right)dy\\ & =\frac{1}{\pi b^{2}}\int_{|y|\leq b}G_{1}+G_{2}\;dy \end{array}$$ (7)

where

$$\begin{array}{c} G_{1}=g\left(t+\sigma_{1},\mu_{1}-y\right)-g\left(t+\sigma_{2},\mu_{1}-y\right)\\ G_{2}=g\left(t+\sigma_{2},\mu_{1}-y\right)-g\left(t+\sigma_{2},\mu_{2}-y\right) \end{array}$$

We approximate *G*_1_ and *G*_2_ by their first order Taylor expansions in time and space, respectively, i.e.

$$\begin{array}{c} G_{1}=A_{1}\left(t,y;\sigma_{1},\sigma_{2},\mu_{1}\right)+R_{1}\left(t,y;\sigma_{1},\sigma_{2},\mu_{1}\right) \end{array}$$ (8)

$$\begin{array}{c} G_{2}=A_{2}\left(t,y;\sigma_{2},\mu_{1},\mu_{2}\right)+R_{2}\left(t,y;\sigma_{2},\mu_{1},\mu_{2}\right) \end{array}$$ (9)

for approximations *A*_1_, *A*_2_ and remainders *R*_1_, *R*_2_. Using this decomposition in Eq (7), we can now show that *v*_1_(*t*, *x*_1_) − *v*_2_(*t*, *x*_2_) decays like a power law of order 2. The approximations have the form

$$\begin{array}{cc} A_{1} & =\left(\sigma_{1}-\sigma_{2}\right)\partial_{t}g\left(t+\sigma_{1},\mu_{1}-y\right)\\ & =(\frac{4D}{{\left(t+\sigma_{1}\right)}^{2}}-\frac{|\mu_{1}{-y|}^{2}}{{\left(t+\sigma_{1}\right)}^{3}})\frac{\sigma_{2}-\sigma_{1}}{16\pi D^{2}}\exp(-\frac{|\mu_{1}{-y|}^{2}}{4D(t+\sigma_{1})}) \end{array}$$ (10)

$$\begin{array}{cc} A_{2} & ={\left(\mu_{1}-\mu_{2}\right)}^{T}\nabla_{x}g\left(t+\sigma_{2},\mu_{1}-y\right)\\ & =\frac{1}{{\left(t+\sigma_{2}\right)}^{2}}\frac{{\left(\mu_{2}-\mu_{1}\right)}^{T}\left(\mu_{1}-y\right)}{8\pi D^{2}}\exp(-\frac{|\mu_{1}{-y|}^{2}}{4D(t+\sigma_{2})}) \end{array}$$ (11)

where *D* = *λ*^2^/*τ*, and the superscript *T* denotes matrix transposition.

In the approximations *A*_1_ and *A*_2_ in (10) and (11), we can identify power laws of order 2 and 3. Hence, for large times, the integral of *A*_1_ + *A*_2_ over the disk |*y*| ≤ *b* goes like 1/*t*^2^. It remains to show that the integral of *R*_1_ + *R*_2_ over this disk has the same or a faster decay. By Taylor’s theorem, there are numbers 0 < |*σ*| < |*σ*_1_ − *σ*_2_| and 0 < *μ* < 1, which in general may depend on *t* and *y*, such that the remainders have the form

$$\begin{array}{cc} R_{1} & =-\frac{{\left(\sigma_{1}-\sigma_{2}\right)}^{2}}{2}\partial_{tt}^{2}g\left(t+\sigma_{1}+\sigma ,\mu_{1}-y\right)\\ R_{2} & =-\frac{1}{2}{\left(\mu_{2}-\mu_{1}\right)}^{T}H\left(\mu_{2}-\mu_{1}\right)\\ & \text{for}\;H=D_{xx}^{2}g\left(t+\sigma_{2},\left(1-\mu \right)\mu_{1}+\mu \mu_{2}-y\right) \end{array}$$

where $D_{xx}^{2}$ denotes the Hessian of *g*(*t* + *σ*_2_, *x*). By direct inspection, *R*_1_ and *R*_2_ can be estimated from above by power laws of order 3 and 2, respectively. Thus, we conclude that the dominant contribution in the decay of *v*_1_(*t*, *x*_1_) − *v*_2_(*t*, *x*_2_) is a power law of order 2. This remains true if the box function *B* degenerates to a point *δ*_*x* = (0,0)_ corresponding to a radius *b* = 0.

#### Simulation of the TMS artifact model

**Fig 3:**
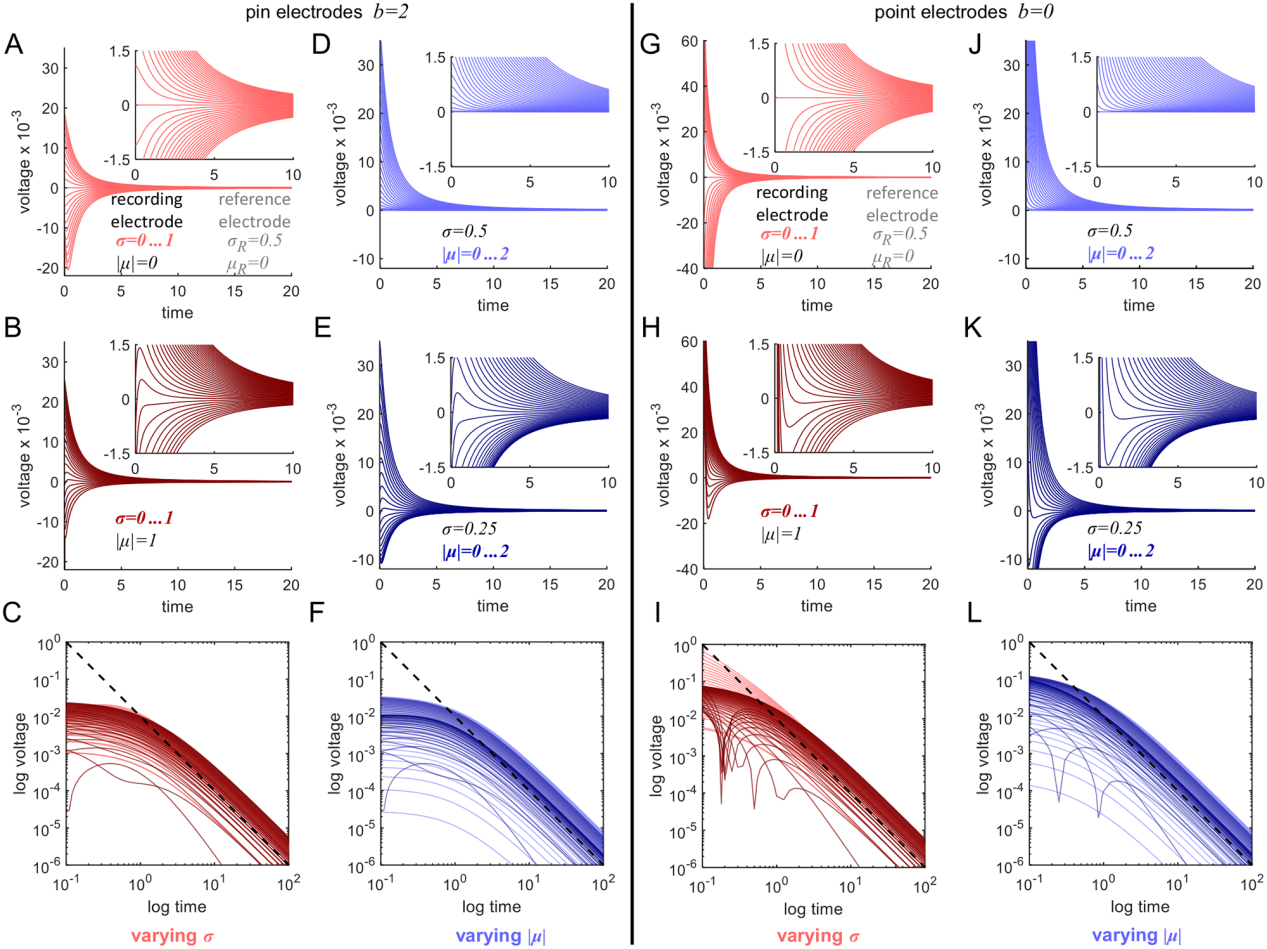
The simulated voltage difference between a reference and the recording electrode. The initial voltage distribution at each electrode is a Gaussian of the form *g*(*σ*, *x* + *μ*) convolved with the box function *B*, where *g*(*t*, *x*) is the voltage impulse-response function of our model, and *B*(*x*) equals 1/*πb*^2^ for |*x*| ≤ *b*. The parameters for the reference electrode are fixed to *σ*_*R*_ = 0.5 and *μ*_*R*_ = 0. The choice of *μ*_*R*_ implies that the voltage depends only on the length |*μ*| of *μ* and not on its direction. The electrode radius was *b* = 2. (A, B) Effect of varying *σ* from 0 to 1 in steps of 0.05 (inset: close-up for *σ* for half step size). (D, E) Effect of varying |*μ*| from 0 to 2 in steps of 0.1 (inset: close-up for |*μ*| for half step size). (C, F) The plot on a log-log scale demonstrates the power law. In comparison to data, we note the asymmetry of positive and negative voltage shapes (compare to Figs 4A, 7A and 11A) and the emergence a local extremum near 0 in some traces (compare to Fig 4A). (G-L) The simulated voltage difference for electrodes approximated by points (*b* = 0).

**Fig 4:**
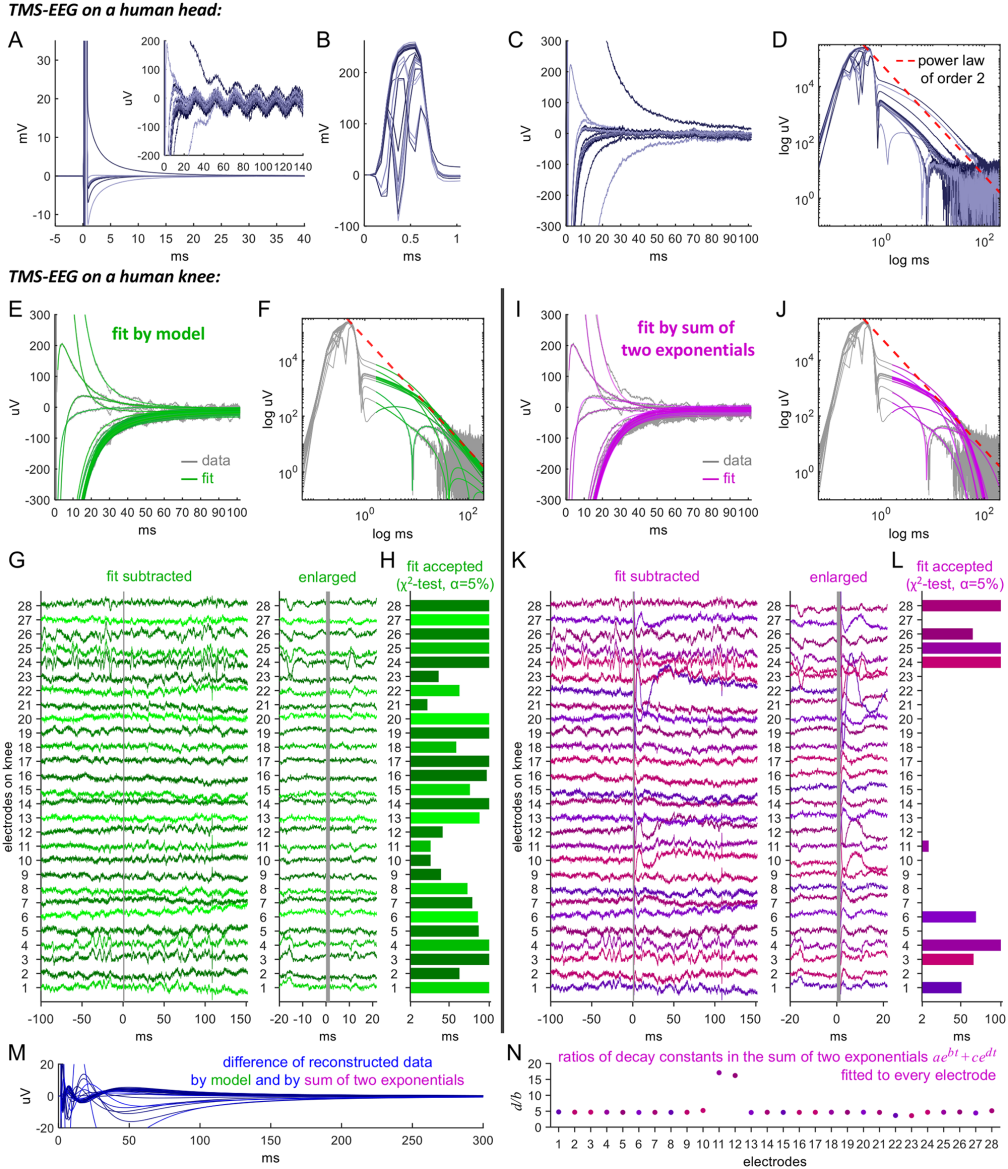
Representative example of TMS artifacts on a human head and assessment of the artifact model. (A) Example of raw EEG traces displaying TMS-induced artifacts recorded from a human head. Magnetic stimulation was applied at time 0 at electrode Cz on a 64-electrode cap with 32 electrodes on the right hemisphere. The artifacts appear in every electrode trace at different strength or shape. Inset: On the uV scale of physiological brain waves, some traces exhibit an artifact duration of more than 100 ms. (B) The fast initial artifact dynamics related to the magnetic pulse. (C) Averaging out noise using five trials shows the long-lasting artifact decay to baseline. (D) On a log-log scale, the tails in the decay of the artifacts (from C) follow a power-law with an exponent on the order of 2 (red dashed lines). (E) TMS-EEG traces on a human knee. Shown are raw data (gray) of a single recording from 28 electrodes covering the knee following TMS. The artifacts reconstructed with the model are shown in green. (F) Log-log plot. (G) Data after subtraction of the reconstructed artifacts followed by subtraction of average of all traces to remove the common mode. As expected from TMS on a knee, the stimulation does not evoke neuronal activity, such that the artifact-removed traces are flat up to continuation of the typical very slow and small electrode drifts. The area shaded in gray indicates where the artifacts could not be reconstructed. (H) To assess the goodness of fit, we use the *χ*^2^-test with significance level *α* = 5%. Shown is the maximal time span for which the test accepts the fit. Beyond this time, the fit is rejected. Small electrode drifts and noise due to TMS-device recharging can shorten this time, however never below 20 ms. (I-J) Fits of sums of two exponentials. (K) Subtraction of the fits introduces both fast and slow distortions of the data in almost all traces. (L) Correspondingly, the fits are generally not accepted, except mainly in noisy and drifting electrodes. (M) The difference of data reconstructed by the model and by the sum of two exponentials. This equals the difference of the respective reconstructed artifacts. (N) For almost all fits by a sum of two exponentials, the quotients of the decay constants are approximately equal and have the same order of magnitude (*a*, *b*, *c*, *d* constants, *t* time).

Fig 3 shows the behavior of the voltage difference versus time as produced by the model. The ‘reference’ electrode that is subtracted from all other electrode voltages was defined by parameters *σ*_*R*_ = 0.5 and *μ*_*R*_ = 0. Numerical simulations of the voltage differences are shown in Fig 3 for recording electrodes characterized by values of *σ* ranging from 0 to 1 and *μ* with length ranging from 0 to 2. Because the displacement *μ*_*R*_ of the reference electrode is 0, *v*_1_(*t*, *x*_1_) − *v*_2_(*t*, *x*_2_) is independent of the direction of *μ* of the recording electrode, and we therefore consider only the length of *μ*. Fig 3A and 3B show the effect of varying *σ* while keeping the displacement length fixed (|*μ*| = 0 for Fig 3A and |*μ*| = 1 for Fig 3B), and Fig 3D and 3E for varying |*μ*| and fixed spread (*σ* = 0.5 for Fig 3D and *σ* = 0.25 for Fig 3E). In Fig 3F–3L, the simulations are repeated for a point approximation of the electrodes’ physical shapes, i.e. *b* = 0 instead of *b* = 2. Furthermore, note the appearance of both positive and negative values of the voltage, which is consistent with measured voltages as seen below in some of the electrodes (compare Figs 4A and 3C). The positive and negative voltages are not necessarily symmetric. Fig 3C and 3F show the voltages on a log-log scale, demonstrating the power-law behavior. It can be seen that the model reproduces very well the full range of the behavior exhibited by the electrodes.

### Technicalities of constructing an artifact removal method based on the model

The model can be used to derive a method for TMS-induced discharge artifact reconstruction. The rationale is to utilize the expressions for the approximate voltage differences between the recording electrodes and the reference electrode and fit them to data. There are two stages in the development of the removal method.

In the first stage we approximate the convolution given in Eq (7) using Eqs (8) and (9). This allows us to use the leading-order approximations *A*_1_ and *A*_2_ as a simplification of the solution. We further approximate *A*_1_, *A*_2_ by their dominant contributions in time. For *A*_1_, the dominant contribution is the sum of two power laws, one of order 2 and one of order 3 (see Eq (10)). For very short times the order-3 power law dominates, while for long times the order-2 power law dominates. For *A*_2_, the dominant contribution is a power law of the form *C*_2_/(*t* + *σ*_1_ + *σ*)^2^, where *σ* = *σ*_2_ − *σ*_1_. We assume that |*σ*| is small and we approximate *A*_2_ further by *C*_2_/(*t* + *σ*_1_)^2^ − 2*σC*_2_/(*t* + *σ*_1_)^3^, where the remainder is an order-4 power law in *t* and hence decays faster in time. Therefore, the dominant contributions to all artifacts can be represented as linear combinations of 1/(*t* + *σ*_1_)^2^ and 1/(*t* + *σ*_1_)^3^ of the form

$$\begin{array}{cc} V & =\frac{v_{1}}{{\left(t+\sigma_{1}\right)}^{2}}+\frac{v_{2}}{{\left(t+\sigma_{1}\right)}^{3}}=\frac{v_{1}t+v_{1}\sigma_{1}+v_{2}}{{\left(t+\sigma_{1}\right)}^{3}} \end{array}$$ (12)

Here, *v*_1_, *v*_2_, and *σ*_1_ are parameters to be determined by fitting. In practice, we replace the expression for *V* by a rational function of the form (*α*_1_ *t* + *α*_0_)/(*t*^3^ + *β*_2_*t*^2^ + *β*_1_*t* + *β*_0_) and impose the additional constraints on *β*_0_, *β*_1_, *β*_2_ to be non-negative. These constraints restrict the parameter space and assure that the denominators have no intercepts for *t* > 0. As a result, singularities in the fitting terms are excluded, as required for *A*_1_ and *A*_2_.

The first stage approximates the artifact at one electrode. In practice it is convenient to implement this fitting procedure on a small subset of electrodes for which artifacts are large compared to the amplitudes of physiological activity (details described below).

In the second stage, we approximate the artifact at any other electrode by a linear combination of the parameters obtained on the ‘large amplitude’ subset. This is visualized in Fig 2C. If the subset of electrodes is suitably selected (typically up to six electrodes suffice) then the initial voltage distributions at the remaining electrodes are all easily accounted for. This simplifies the artifact removal procedure enormously, and is an important consequence of the fact that our model is linear in the initial voltage distribution. Indeed, it implies that the artifacts in all electrodes can be approximated by linear combinations of the fitted artifacts from the electrodes in the subset.

To summarize, our method includes the following steps:

Step 1 Select a subset of *N* = *N*_*p*_ + *N*_*n*_ electrodes chosen such that it consists of the *N*_*p*_ electrodes with the largest positive amplitude deflection after the TMS pulse and another *N*_*n*_ electrodes with the most negative deflection. Generally, we used *N*_*p*_ = *N*_*n*_ = 3. Apply the fitting procedure to each of these electrodes to obtain the parameters *α*_0_, *α*_1_ and *β*_0_, *β*_1_, *β*_2_ that reconstruct the corresponding discharge artifacts.
Step 2 Project every electrode onto the *N*-dimensional space spanned by the discharge artifacts reconstructed in Step 1.

Fitting is performed over a specified time range, such as from 8 ms to 28 ms for a sampling rate of 1 kHz, and from 1.5 ms to 25 ms for 8 kHz. This is the time it typically takes the largest positive and negative artifacts to decay back to physiological range of ±100 μV. If the data includes muscle artifacts exceeding ±500 μV, the time range may need manual adaptation, but in general the algorithm runs automatically. The algorithm was developed using Matlab’s Optimization Toolbox. A constrained least-squares algorithm was used for Step 1. Finally, the artifact-free data are obtained by subtraction of the artifact fits.

### Experimentally testable implications and consistency checks of the model

To validate the model and demonstrate its ability to account for the discharge artifacts, we identify seven different implications and two consistency checks that can be directly tested using real TMS-EEG data.

#### Implication 1: The voltage decay of the artifact exhibits an order-2 power law tail

In the above formulation of the model we described the presence of several decay regimes and how an initial 1/*t* dynamics can be obtained via spatial diffusion. In addition, we demonstrate that while the power law of the discharge voltage versus ground is of order 1, discharge voltage versus reference is of order 2. The presence of this power law decay regime is a hallmark of our model, departing from the current view that the decay is exponential [3, 5, 17, 19, 20]. Although the view of the skin as a resistive-capacitive layer is well-established, it is commonly modeled by a single leaky capacitor (see [6]), which is characterized by an exponential decay and cannot reproduce a power law.

#### Implication 2: TMS-EEG on human skin leads to order-2 power law artifact decay, whereas non-skin surface structures typically deviate from this decay

A fundamental ingredient of our model is the structure of human skin, which gives rise to the order-2 power law in the artifact decay. Applying TMS-EEG on different surface structures will give different decay dynamics. Stimulation of the human knee is one case that is expected to have similar conduction properties to the human scalp. Since TMS does not typically stimulate ongoing neuronal activity in the knee, we expect to be able to fully reconstruct the artifacts in EEG-TMS from the knee. In this setting the natural EEG data consists of tonic muscle activity and of small, slow drifts in some electrodes. If the model or the associated fit have any systematic errors, then their subtraction from the knee data would result in residual artifacts. Otherwise, if the fit is good, then subtraction of the reconstructed artifacts from the EEG-TMS knee data will be featureless when compared to brain EEG data.

Previous work compared human TMS-EEG artifacts to data acquired from phantom heads, using a muskmelon [26, 27] and a watermelon [28]. It is an implication of our model that, due to fundamentally different the surface properties compared to the scalp, a substantial deviation from the power law decay dynamics is observed.

#### Implication 3: Skin preparation (puncturing and exfoliation) reduces TMS artifact amplitudes

Since the artifact in our model is attributed to properties of the skin, the artifact decay should depend on specific dermal properties. The resistance of the skin is on the order of several tens of kiloohms and exhibits a complicated frequency dependence [7, 29], with the largest contribution by far coming from the stratum corneum. If this layer is removed or punctured, the resistance drops to a few hundred ohms [7, 8, 30]. This effect of puncturing underneath recording electrodes attached to the skin was first reported almost a century ago [31] and is a known method for motion artifact reduction in EEG [32] and in EEG-TMS [10]. In our model of the skin as a resistive-capacitive layer, this locally decreases the resistance and possibly the capacitance of the gel-skin interface. Skin preparation as commonly applied to decrease electrode resistance in passive EEG systems is usually performed by skin exfoliation (“rubbing”) under the electrodes or by delicate puncturing with a needle of the epidermis. According to our model, if TMS is applied before and after reduction of skin preparation, a substantial change in artifact decay is expected to occur. One implication is that skin preparation techniques affects the amplitude of the discharge artifacts but also that a transition to a power law decay still occurs. The duration of the power law under these conditions is not an obvious implication.

#### Implication 4: Positioning of the reference electrode w.r.t. the coil and to the recording electrodes has direct implications on the power law of the decay

Since the measured voltage at a recording electrode is subtracted by the reference electrode, and both are affected by artifact dynamics, the artifact shape will be influenced by the relative position of recording and reference electrodes. Moving the reference electrode away from the coil will also change the subtracted shape. If the position of the reference electrode is changed while the stimulation site, the head position, and the locations of the recording electrodes are kept fixed then substantial alteration in the shape of the artifacts will be observed.

#### Implication 5: Artifact power law decay is not affected by the sampling rate

In the acquisition and processing of fast pulse and decay processes, we must consider hardware effects of the EEG amplifier such as the bandwidth and slew rate. The sampling rate determines the precision and possible distortion of the recording of the actual artifact shapes (e.g., distortions of around 10 ms after the pulse can occur for an acquisition rate of 1024 Hz compared to 16384 Hz). Our model implies that these short time scales will not change the order-2 power law in the artifact decay tails.

#### Implication 6: Artifact power law decay is not affected by the amplifier design

The model does not depend on aspects of the amplifier design, such as the reliance on active electrodes. Active systems incorporate an additional pre-amplification stage directly at each electrode, whereas electrode in passive systems are simple conductors. The discharge artifact dynamics can therefore be expected not to be affected.

#### Implication 7: Discharge artifacts can be separated from TMS-activated muscle artifacts

The model posits that the artifacts arise from relaxation of the skin capacitor. TMS-pulse application can, however, additionally activate cranial muscles. The artifacts are therefore expected to be different from muscle artifacts, in shape and in temporal dynamics.

#### Consistency test 1: Reconstruction of TMS recharge and power line artifacts

We check whether the artifact removal method based on our model can reconstruct several technical features of the EEG signal. The first is the TMS recharge artifact, which is a characteristic of the Magstim power supplies that we use to operate the magnetic coil [27]. The second technical signal that we reconstruct is the 50 Hz pickup from the power line signals.

#### Consistency test 2: Reconstruction of TMS-evoked potentials by motor cortex stimulation

In the literature, it is reported that evoked potentials can be observed in EEG after a TMS pulse is delivered to the motor cortex [4]. We can therefore verify that our model-based artifact removal method can reconstruct the reported potentials.

### Experimental conditions for testing the implications

Here we summarize the details for the experiments for testing each of the implications and consistency checks listed above.

#### Testing Implications 1 and 2: Stimulation of human head, human knee and phantom heads

The human-head EEG data for Fig 4A–4D were recorded from a single subject fitted with a 64-electrode cap of which only the 32 electrodes on the right hemisphere were connected. Acquisition rate was 16384 Hz and the stimulation site was Cz. The human-knee EEG data for Fig 4E–4L were recorded from a single subject at 16384 Hz. The knee was held in a bent position and covered with a 64-electrode cap with 30 electrodes connected (2 were subsequently removed from analysis due to strong 50 Hz noise). The cap was positioned such that the CPz electrode was located over the center of the kneecap. TMS stimulation was on CPz with the coil positioned about one cm above CPz at stimulation strength of 80%.

**Fig 5:**
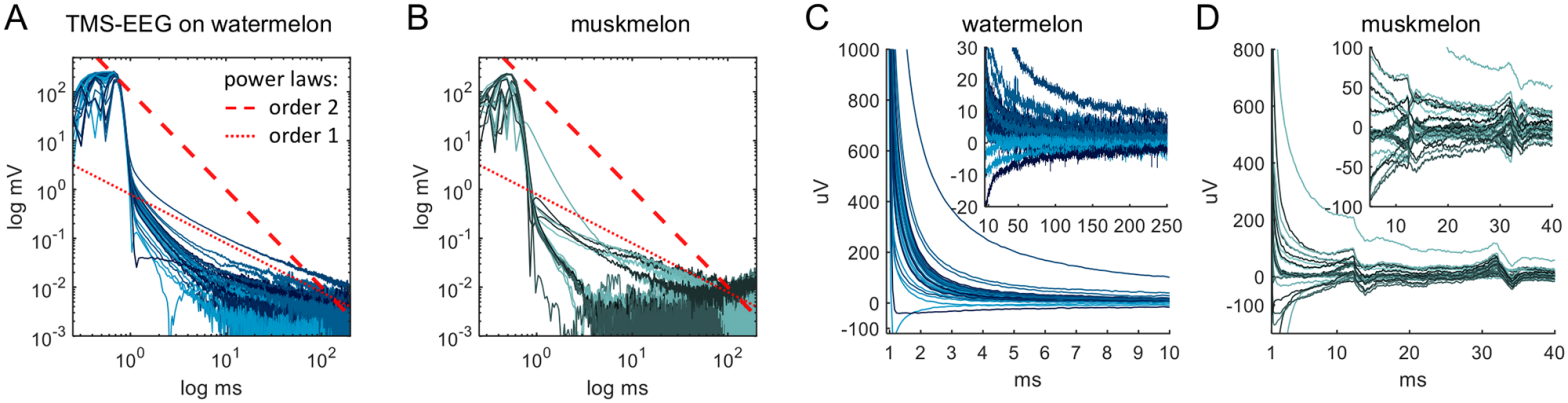
TMS-EEG on phantom heads with non-human skin structure. (A, B) Log-log plots of TMS artifacts on a watermelon and on a muskmelon show artifact decay is very different from a human head in that it does not follow a power law of order 1 (dotted line) or 2 (dashed). (C) The artifact traces on the phantom qualitatively resemble the artifact on a human head even though they are much smaller and generally shorter-lasting. (D) Example of an artifact which contains an additional recharging artifact consisting of two waves at an interval of 20 ms, where the first wave appears within around 20 ms after the pulse. This artifact can appear when the TMS device is operated with two boosters. It is visible in around half of all trials at different intensities and is not affected by acquisition rate. Inset: Subtraction of the common average from the traces turns the recharging artifact into two ‘blips’.

We used a watermelon and a muskmelon (cantaloupe) as phantom heads and recorded from 32 electrodes located on one hemisphere of the 64-electrode EEG cap at 16384 Hz. In order to obtain a meaningful log-log plot of the trial average for every electrode, care was taken to avoid electrodes with drifts contributing a substantial non-zero offset. To this end, if an electrode trace drifted in a trial out of the range of ±40 μV during 1.5 s to 2 s after TMS pulse application, the electrode of this particular trial was excluded from averaging. The mean number of electrodes removed per trial for the watermelon was 6.84 but no electrode was removed from all trials. A total of 61 trials were averaged. For the muskmelon the signals were of much lower quality, so that 9 electrodes were removed for all trials. From the remaining 23 electrodes, the mean number of electrodes removed per trial was 16.46. A total of 63 trials were averaged for the muskmelon. Fig 5D shows a single muskmelon trial with a strongly visible TMS device recharge artifact. Stimulation site for the phantom heads was at Cz.

#### Testing Implication 3: Skin puncturing and exfoliation

**Fig 6:**
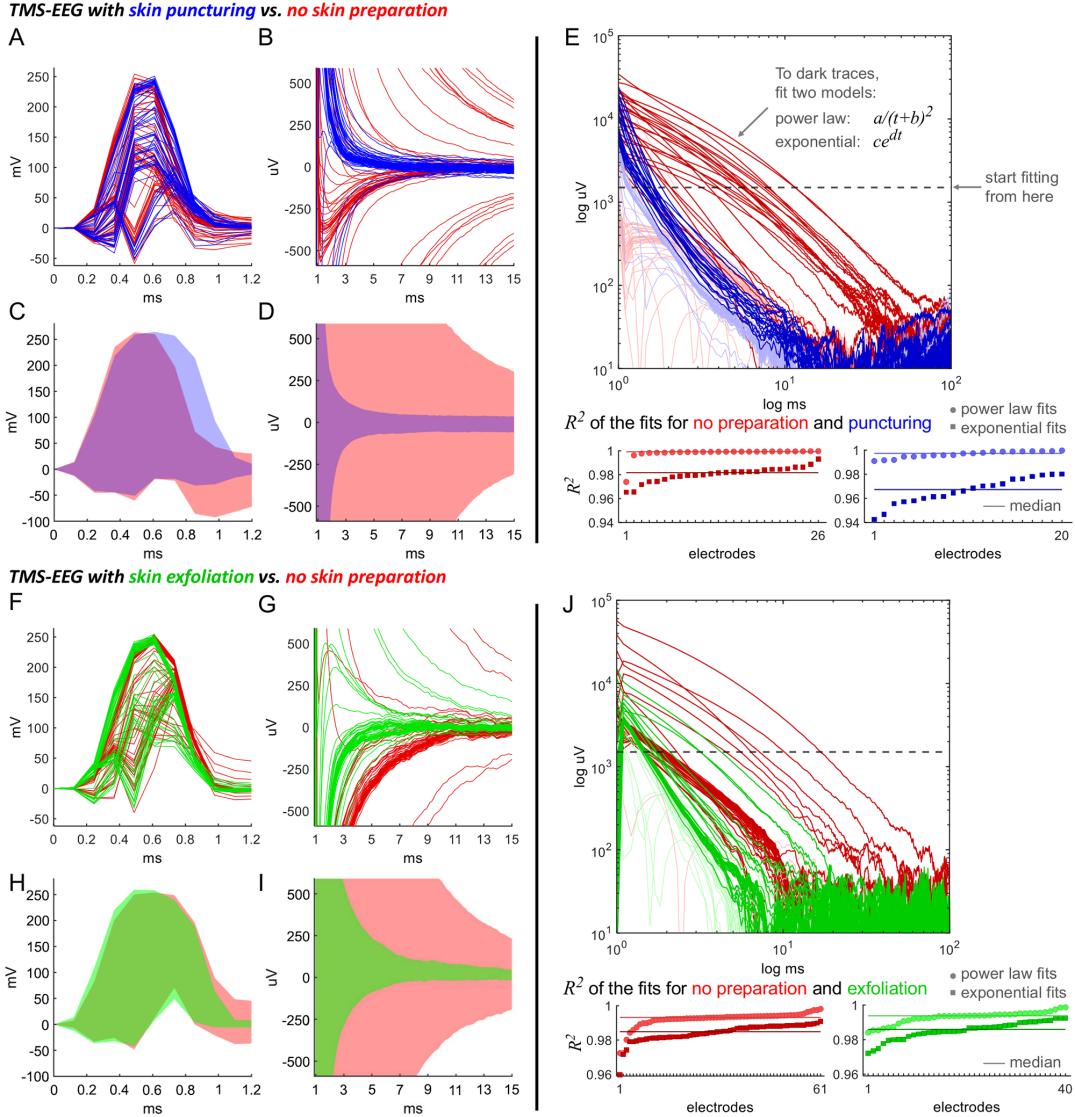
The TMS-induced artifacts before and after skin preparation by puncturing and exfoliation. (A, B) Sample of raw EEG traces from a 64-electrodes cap after TMS application at CPz at time 0. Acquisition rate was 8 kHz to resolve the fast initial artifact dynamics. The recording before (red) and after skin puncture underneath the EEG electrodes (blue) shows no difference in the dynamics of the pulse artifact (A) but a reduction of the artifact decay (B). (C, D) Shown is the envelope (shaded area) of the two distributions of all artifacts before and after puncture. These distributions of artifacts were obtained by combining all sets of 64-electrodes cap traces from 2 subjects, each stimulated at both CPz and CP3 in a total of 71 TMS pulses. The pulse artifact is not changed (within an accuracy of one time step) by skin puncturing (C). The amplitude of the decay artifact (D) is reduced as shown by the shaded area, corresponding to the 5%-to-95% percentile of the distribution of all artifacts. (E) We compare two physical models of decay, (shifted) power laws *a*/(*t* + *b*)^2^ and exponentials *c*exp(−*dt*) (*t* time, *a*, *b*, *c*, *d* constants). Both models are least-squares fitted to all traces which do not change sign and have amplitude larger than 1.5 mV (dashed line). All fits are done to 25 ms starting from the point of reaching 1.5 mV. Evaluation of the fits by *R*^2^ shows the power law is better than the exponential with and without skin treatment. Specifically, skin puncturing does not decrease the difference of *R*^2^ by median (solid lines). (F-J) Same as (A-E) with skin exfoliation (green) instead of skin puncturing compared to control (red). Stimulation site was Cz and FP2, sampling rate 8 kHz.

To assess the effect of changes in skin impedance (see Fig 6 below) we conducted experiments with punctured or exfoliated skin. For puncturing, the skin under each electrode (including the DRL and CMS electrodes) was pierced twice, following the protocol described in [10]. Briefly, a 25-gauge needle was put in a small pipette tip such that only 0.5 mm of the needle tip extended beyond the pipette tip. The puncturing was applied following head cap placement and gel application. Skin exfoliation was performed by rubbing the skin with an exfoliation paste provided by g.tec. For skin puncturing, data were collected from two subjects totaling 71 trials (40 control and 31 with skin puncture). Stimulation sites were at CP3 and CPz. For skin exfoliation, data were collected from a single subject totaling 20 trials (5 trials for each condition). Stimulation sites were at Cz and FP2. Envelopes of all the trials together are presented, in which we took all the measured points from all trials and depict them as a solid area. Outliers were avoided in these trial envelopes by discarding the highest and lowest 5% points of the traces.

#### Testing Implication 4: Change of reference electrode placement

**Fig 7:**
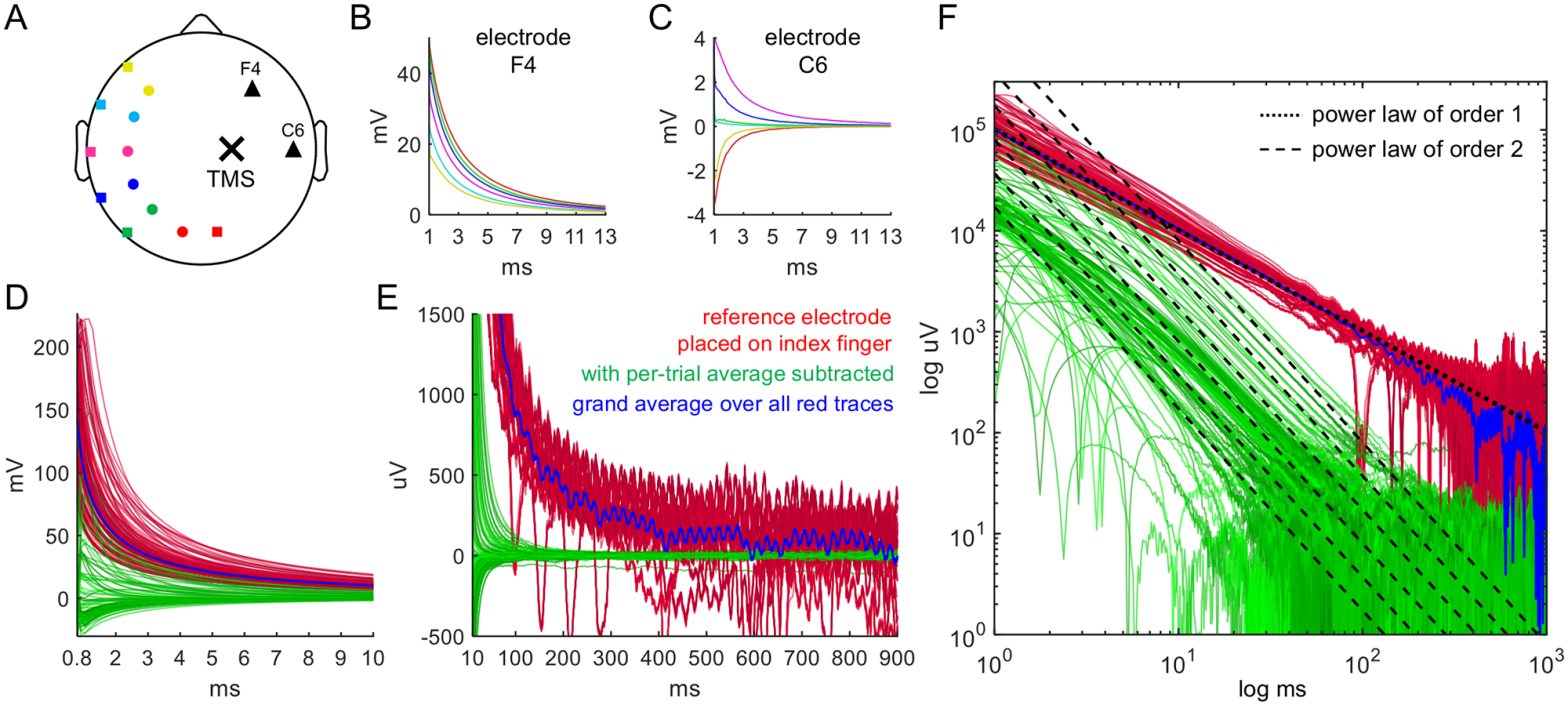
Effect of placing the CMS and DRL electrodes at different locations relative to the TMS coil. (A, B, C) Recordings from electrodes F4 and C6 (triangles) for TMS application at C2 (cross) are shown for different placements of the CMS (circles) and DRL (squares) electrodes. Different colors correspond to different placements of CMS-DRL (red = standard positions assigned on the Biosemi cap, green = P3-P7, blue = CP3-TP7, pink = C3-T7, cyan = FC3-FT7, yellow = F3-F7). (D) EEG traces after TMS stimulation at time 0 with the CMS and DRL electrodes placed on the index finger of the left hand. The corresponding electrode wires had a minimal distance of 50 cm from the TMS coil. The points of stimulation consisted of Cz, CPz, and POz. Shown are 13 trials with 13 electrode traces each acquired at 16 kHz. The red traces correspond to the raw, unmodified electrode traces. The green traces correspond to the same data where in addition, in every trial, the average of all 13 electrodes was subtracted from the data. Because the raw traces exhibit strong 50 Hz noise due to the unusual ground electrode placement, the grand average of the red traces over all trials is also shown (blue trace). (E) Same data on a longer time scale. Note the similarity of the variety of the green traces to the artifacts in Fig 4A. (F) On a log-log scale, the red follow a power-law with an exponent on the order of 1 (black dotted line). The green traces follow a power-law upon late-stage decay to baseline with an exponent on the order of 2 (black dashed lines).

In order to investigate the effect of reference electrode placement, the EEG data for Fig 7A–7C were recorded from a single subject with 32 electrodes located on the right hemisphere of the 64-electrode EEG cap. Sampling rate was at 16384 Hz. Trials were recorded with CMS and DRL their original positions on the Biosemi cap, relocated to P3 and P7, to CP3 and TP7, to C3 and T7, to FC3 and FT7, to F3 and F7. Stimulation was at C2.

To examine the effect of reducing the magnetic pulse on the CMS, DRL electrodes and their corresponding wires, we placed the DRL and CMS electrodes on the index finger of the left hand, keeping the distance to the coil at least 50cm. The corresponding EEG data are shown in Fig 7D–7F and were recorded from a single subject with 13 electrodes distributed such that they uniformly covered the whole 64-electrode head cap. Acquisition rate was 16384 Hz. Stimulation sites were Cz, CPz, and POz. Similarly, we examined the effect of eliminating the common-mode rejection by the DRL electrode by bridging DRL and CMS within the same gel blob. As this procedure leads to strong 50 Hz noise, we subtracted from each electrode trace a 50 Hz sine wave with the phase and amplitude obtained from the averaged 50 Hz noise. While this did not completely eliminate the 50 Hz noise, it did reduce it by a factor of 10.

#### Testing Implication 5: Effect of the sampling rate

**Fig 8:**
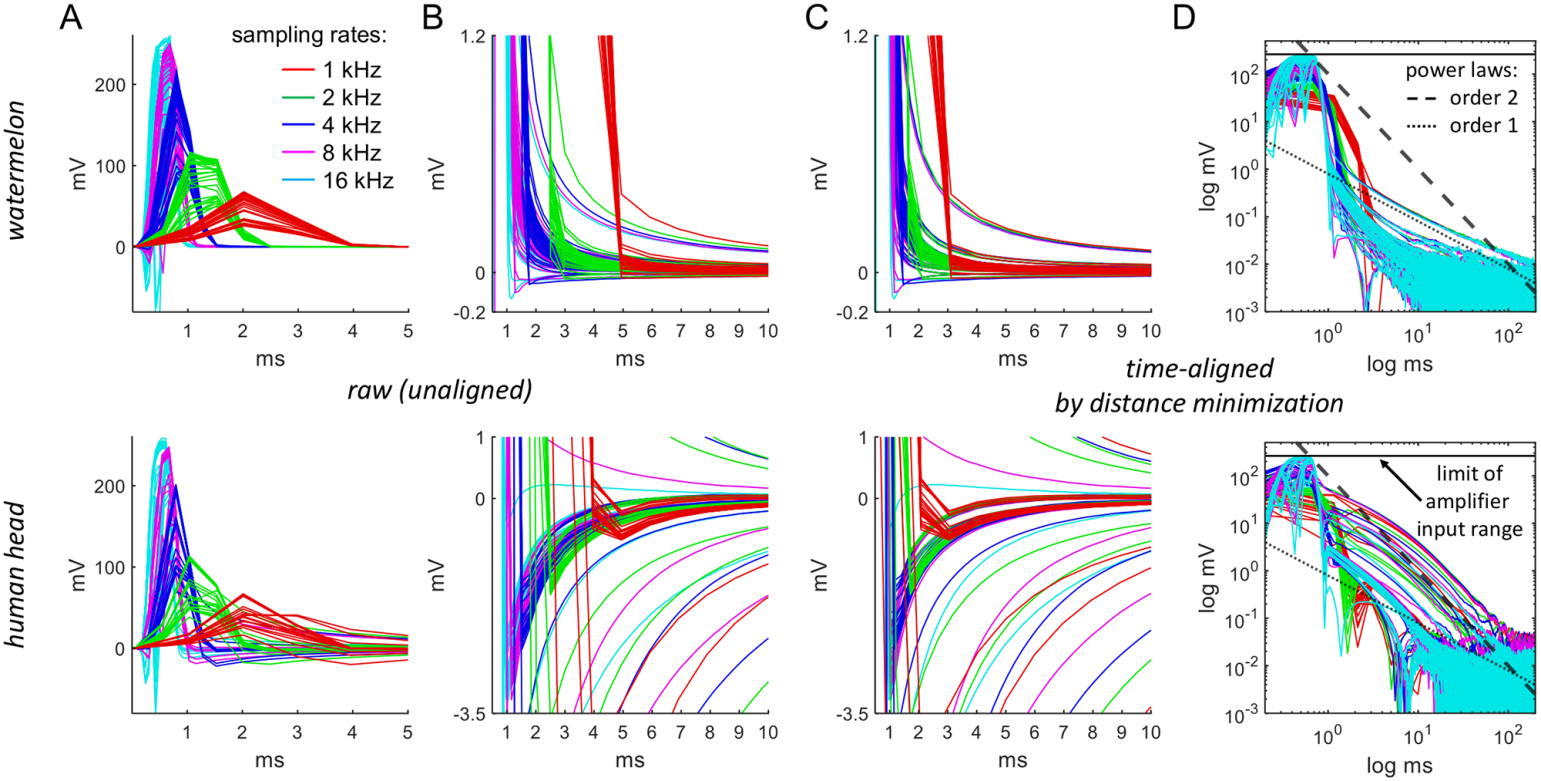
Effect of changing the sampling rate. (A, B) Decreasing acquisition rate (16384 Hz, 8192 Hz, 4096 Hz, 2048 Hz, 1024 Hz) lead to a progressive time shift of the TMS artifacts and a decrease of TMS pulse artifact amplitude. The shifting time can be found by time-shifting the traces for each sampling rate backwards until they coincide with the 16 kHz traces. The optimal time is found when the sum of distances between these traces, evaluated directly after the pulse artifact, becomes minimal. The optimal times coincide for the watermelon and the human head (C). Note that time shifting will not change power law decay tails as can also be seen in (D).

We recorded TMS-EEG from a watermelon and a human head at 1024 Hz, 2048 Hz, 4096 Hz, 8192 Hz, 16384 Hz, as shown in Fig 8 and described for Implications 1 and 2. For all sampling rates, 61 trials were used, and the mean number of electrodes removed per trial was 6.84. The human-head EEG data shown in Fig 8 were recorded from 32 electrodes on the right hemisphere at the different sampling rates in 5 trials and then averaged. Stimulation site for the human and phantom heads was at Cz.

#### Testing Implication 6: Passive versus active EEG systems

**Fig 9:**
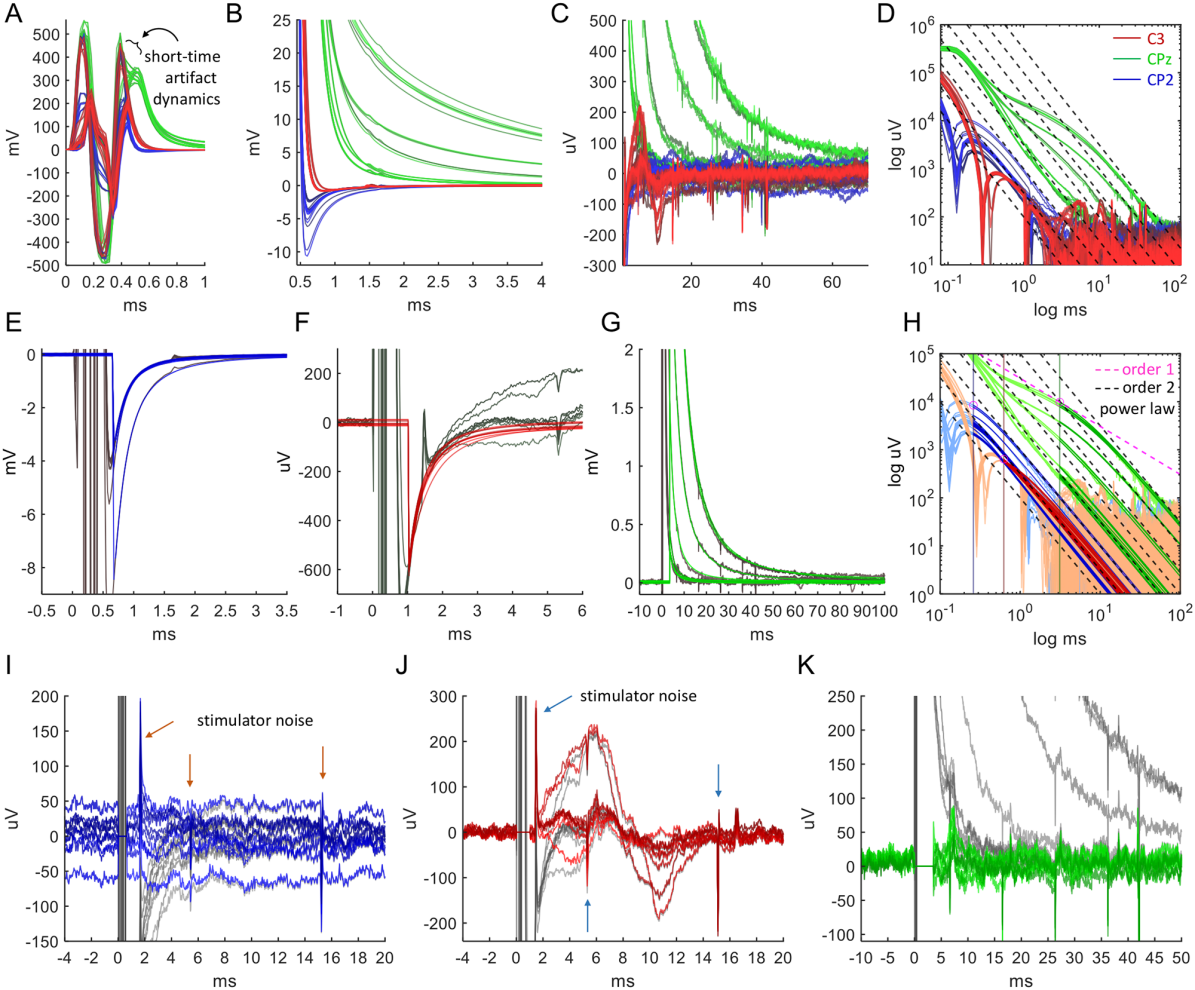
TMS artifacts of a passive EEG system (g.tec) with ring electrodes. Traces were recorded from nine electrodes (F2, F5, F6, C3, C4, CZ, CP5, P5, POz) at 38,4 kHz on a single subject. TMS was applied at time 0. Stimulation sites were C3 (red traces, 4 trials), CPz (green, 3 trials), and CP2 (blue, 3 trials). (A) The TMS pulse artifact is well-resolved due to the high acquisition rate and has a duration of 0.39 ms. Note the short-time artifact dynamics visible in the green traces, which is different from what can be seen in the Biosemi artifacts. (B, C) The skin-capacitor discharge artifacts for a short time (B) and long time (C). (D) On a log-log scale, the late-stage decay of all artifacts exhibits a power law of order 2 like in the Biosemi EEG system. (E, F, G) Single trials with reconstructed artifact fits overlaid on the artefactual data (gray) for each stimulation site as indicated by color. (H) Log-log plot of the artifact fits for all trials overlaid on the data. The first 15 samples corresponding to 0.39 ms containing the TMS pulse were omitted. For each stimulation site the starting time point for the fit is selected as the first point at which all the artifacts in all electrodes already decay faster than a power law of order 1. This is actually the place where a power law of order 1 (magenta) is tangential to the slowest decaying artifact. These time points are marked by vertical lines that correspond to each stimulation site (for CP2 0.31 ms, C3 at 0.63 ms, CPz at 3.12 ms following the end of the TMS pulse). (I, J, K) Reconstructed data by subtraction of the artifact fits. Note that the noise in the form of spikes (arrows in I, J; also visible in E, F), possibly from the TMS stimulator, is reconstructed without distortion.

The EEG data were recorded from a single subject with 9 electrodes at 38400 Hz by a passive EEG system (see Fig 9 and the section on EEG data acquisition below). Skin exfoliation was performed. The stimulation sites were C3, CPz, and CP2.

#### Testing Implication 7: Separation of discharge and muscle-activation artifacts

**Fig 10:**
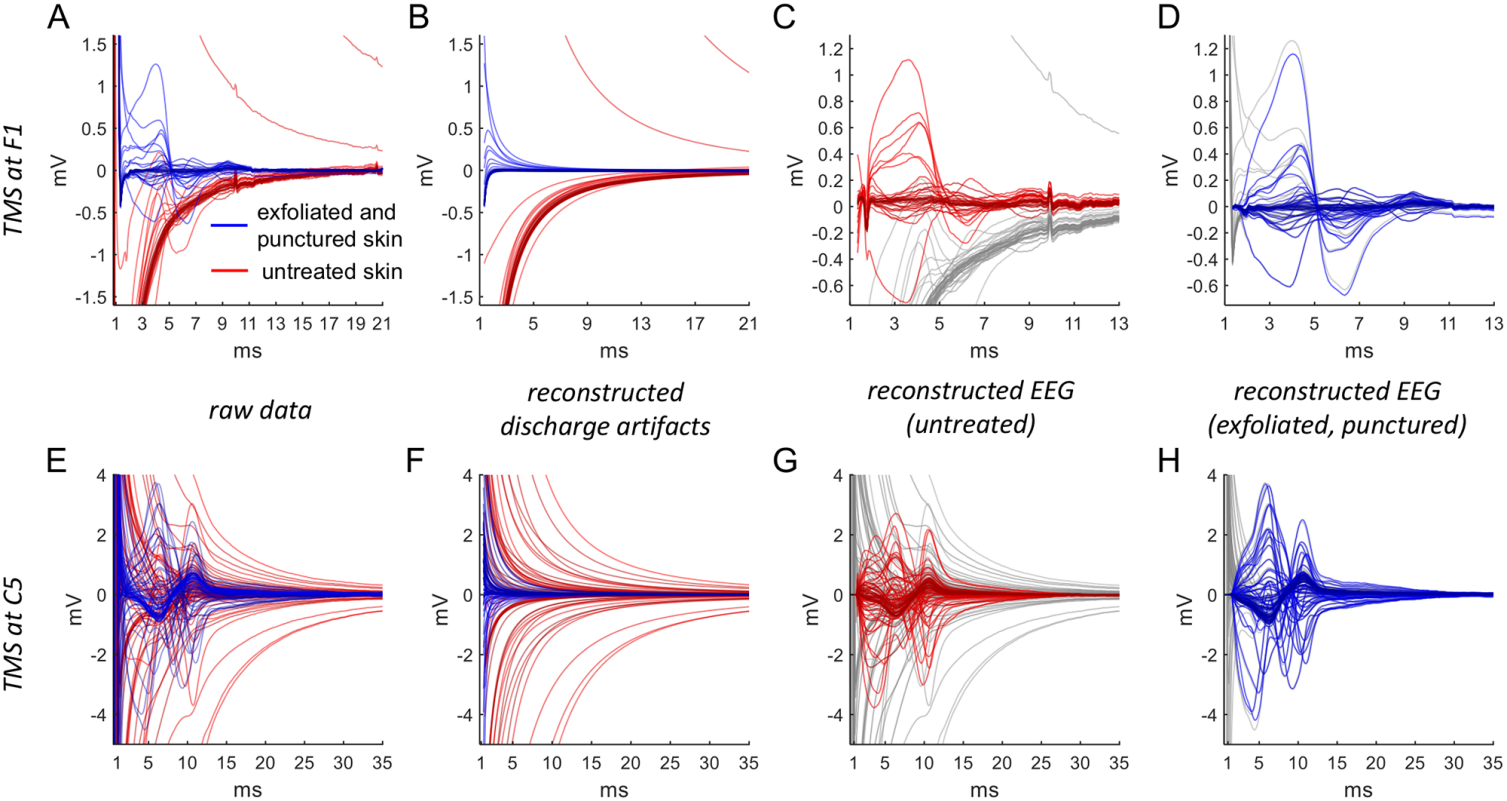
Cranial muscle activation by TMS. Cranial muscle activation experiments were performed with untreated skin (red traces) and, to assess artifact reconstruction, both punctured and exfoliated skin (blue traces) to downscale the TMS discharge artifacts. TMS stimulation was applied at F1 (A-D, subject 1, 32 electrodes) and C5 (E-H, subject 2, 64 electrodes). Sampling rate was 8 kHz, except for stimulation at F1 for untreated skin (C), where it was 16 kHz. The muscle artifacts have amplitudes of up to 4 mV and a duration of up to a few tens of milliseconds. Their biphasic dynamics fall within the first 7 ms (C, D) and 13 ms (G, H).

Experiments in which TMS was used to activate cranial muscles were performed in two conditions. In the first the skin was untreated and in the second the skin was both exfoliated and punctured. The EEG data for Fig 10 were recorded from two subjects. Stimulation was applied at F1 (Fig 10A–10D, subject 1, 32 electrodes) and C5 (Fig 10E–10H, subject 2, 64 electrodes). The sampling rate was 8192 Hz, except for stimulation with untreated skin in Fig 10C, where it was 16384 Hz.

#### Performing consistency test 1: Reconstruction of recharge and power line artifacts

**Fig 11:**
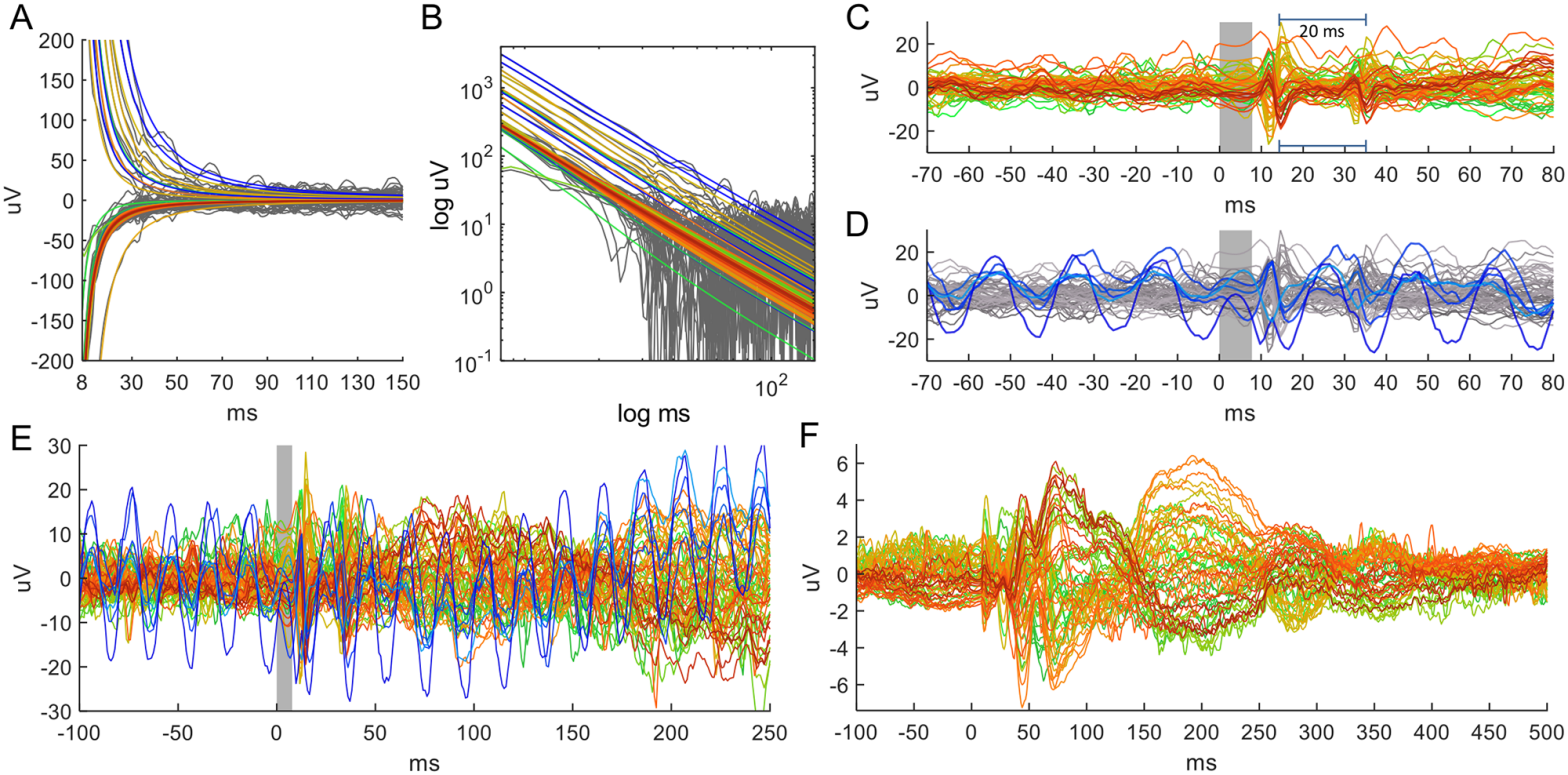
Computationally reconstructed artifacts for given EEG data from a 64-electrodes cap recorded at 1024 Hz. TMS application at FC1. Two electrodes with strong drifts were removed, all data average-referenced. (A) The artifact fits (colored) and original EEG traces (gray). (B) Log-log plot of A. (C) The same sample, still unfiltered, with the artifact fits subtracted. At this low sampling rate the sample points between time of stimulation (origin) and 7.8 ms afterwards could not be reconstructed and are therefore interpolated with splines (shaded in gray). The traces with strong 50 Hz noise (corresponding to 5 electrodes) are not shown (but see D). Note the two spiking recharging artifacts of the TMS stimulator occurring with an exact timing of 20 ms (bars) also found on the phantom head. (D) The five 50 Hz affected traces (blue, the remaining signals are grey). The subtraction of the artifact fit reconstructs the 50 Hz signal corroborating the performance of the reconstruction. Note that recharging artifacts of the TMS stimulator are aligned with the 50 Hz signal. (E) The full sample on a longer time scale high-pass filtered at 1 Hz. (F) Trial average over 99 trials. All trials were additionally low-pass at 300 Hz and notch-filtered at 50 Hz. The brain response to TMS stimulation manifests as TMS-evoked potentials continuing over several hundred milliseconds.

EEG data for Fig 11 were recorded from a single subject with 64 electrodes (2 electrodes removed due to drift) at 1024 Hz sampling rate. The stimulation site was FC1. Due to the presence of a strong common-mode signal, all EEG data in this figure are shown after subtraction of the average of all signals.

#### Performing consistency test 2: Reconstruction of TMS-evoked potentials

**Fig 12:**
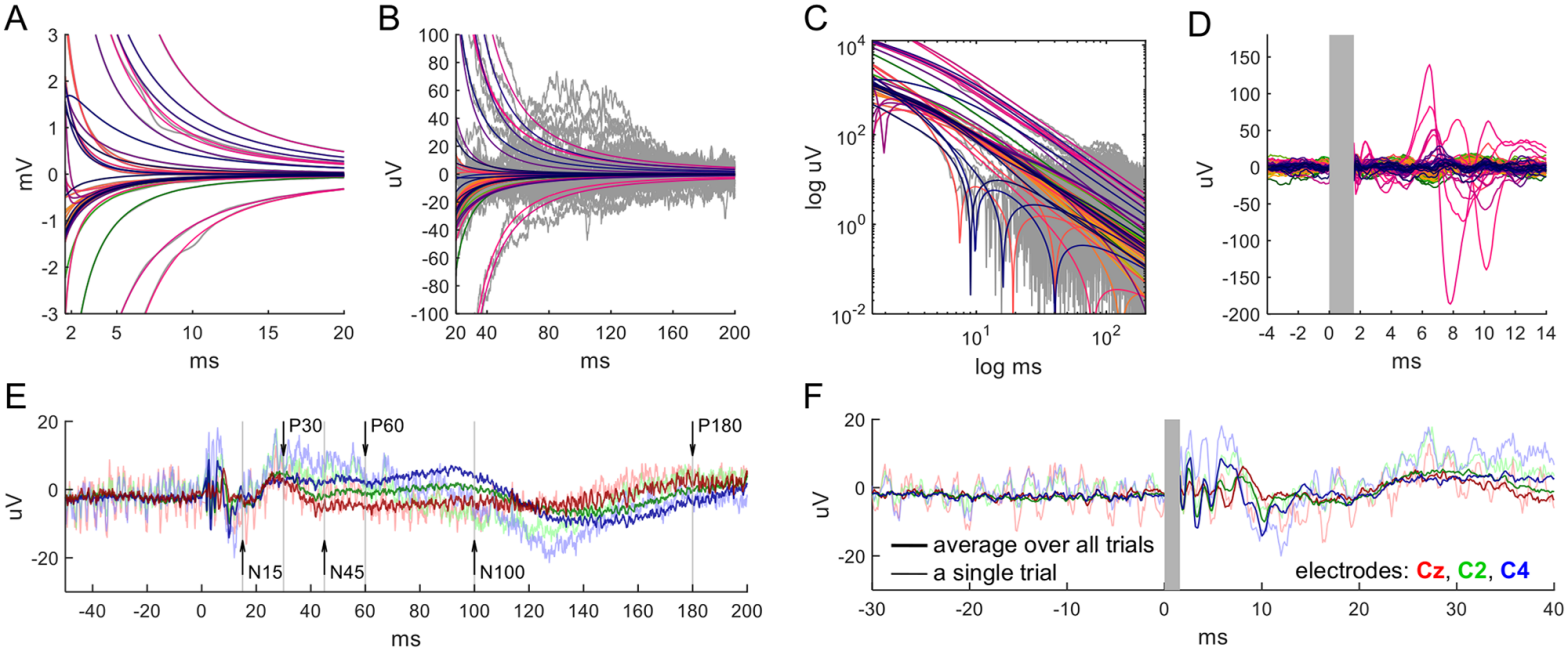
TMS-evoked responses due to stimulation of the right motor cortex. (A) Artifact fits (colored) to data (gray) of a single trial. (B) The fitted artifacts and data on a longer time scale. (C) Log-log plot of the artifact fits. (D) The same data with the artifact fits subtracted. The first 1.6 ms, for which data could not be reconstructed, was marked as gray area. (E-F) Electrodes Cz (red), C2 (green), C4 (blue) of the filtered single trial and of the average over 20 filtered trials (thin and thick lines, respectively). Filtering: 1 Hz high-pass, 50 Hz notch. TMS-evoked potentials as reported in the literature are marked at 15, 30, 45, 60, 100, and 180 ms. All data are average-referenced.

To study TMS-evoked potentials similar to those reported in [4], the right motor cortex of one subject was stimulated by positioning the TMS coil between C2 and C4, slightly shifted towards the frontal direction. The TMS stimulation strength was adjusted such that twitching of the index finger of the left hand could be visually observed. Twenty trials with obvious finger twitches were taken for analysis. These EEG data are presented Fig 12 and were recorded with an acquisition rate of 8 kHz.

### Ethics statement and subjects

Experiments were approved by the Ethics Committee of the Interdisciplinary Center (IDC) Herzliya and by the Sha’ar Menashe Hospital (Israel) internal review committee. All data except those used in Fig 11 were obtained by self-experimentation of E.M. and N.L.B, as approved by the Ethics Committee of the Interdisciplinary Center (IDC) Herzliya. No dependence of any kind (financial, hierarchical, etc.) exists with respect to other authors on the study. The data set used in Fig 11 was obtained from a healthy subject who participated as part of the healthy control group of an experiment which was approved by the Sha’ar Menashe Hospital (Israel) Internal Review Board. All participants provided their written consent to participate in this study.

### EEG systems and recordings

EEG recordings were obtained using the ActiveTwo (Biosemi, Netherlands), which incorporates a DC-coupled amplifier with 24-bit resolution. The electrodes used were pin-type active Ag/AgCl electrodes with a Common Mode Sense (CMS) electrode and a Driven Right Leg (DRL) electrode. In this configuration, instead of the usual reference electrode there is a CMS electrode to sense the common mode signal and a DRL electrode which injects back the common mode signal, thus rejecting the common mode for all electrodes. The electrodes were connected to the scalp using a head cap (standard Biosemi 64-electrode head cap) and electrode gel (Signa Gel 15-25, Parker, USA). The CMS electrode in this cap is located between P1, Pz, PO3 and POz and DRL is between P2, Pz, PO4 and POz.

To inspect the TMS artifacts in EEG for a passive EEG system, we used the g.USBamp (g.tec, Austria) in passive recording mode (see Fig 9). This system also uses a DC-coupled amplifier with 24-bit resolution. Electrodes were the g.SCARABEO ring electrodes. The electrodes were connected to the skin using the g.GAMMAcap and the same gel as for the ActiveTwo experiments. The reference and ground electrodes were placed on the left mastoid and forehead, respectively. Since we worked in the passive mode, the skin was exfoliated before gel application in order to reduce skin resistance.

### EEG data acquisition

Acquisition rates for the Biosemi ActiveTwo system were 1024, 2048, 4096, 8192, and 16384 Hz. The maximal number of electrodes depends on the acquisition rate, with a possibility of using 32 electrodes for 16 kHz, 64 for 8 kHz, etc. up to 256 electrodes. The acquisition rate affects the amplifier bandwidth (for the ActiveTwo, the maximal bandwidth is 400, 800, 1600 and 3200 Hz at sampling rates of 2048, 4096, 8192 and 16384 Hz, respectively) which in turn affects the resolution of the initial sharp peak of the recorded TMS artifacts. All EEG traces were offset-corrected by subtracting their time-average over 1 s. All presented EEG data are raw (and offset-corrected), i.e. no common average was subtracted unless explicitly mentioned. Likewise, no additional filtering was applied, unless explicitly mentioned. Where filtering was performed, the eegfiltnew function of the EEGlab toolbox for Matlab was used (Release 2017a, The MathWorks, Inc., USA).

The acquisition rate for the g.tec system was 38400 Hz in passive recording mode. Recording was from 9 electrodes located at F2, F5, F6, C3, C4, Cz, CP5, P5, POz. Because the TMS artifacts recorded with the g.tec system were very short (Fig 9) and hence buried within 50 Hz noise, for this data we removed the 50 Hz noise by fitting 50 Hz sine waves to each baseline EEG trace and subtracted them.

### TMS stimulation device

The Magstim Rapid (The Magstim Company, UK) was used for TMS pulse application, which was delivered by a standard 7 cm figure-of-eight coil. The magnetic pulse waveform is a single sine cycle inducing a current waveform of a single cosine cycle. Although the MagStim Rapid manual reports a pulse duration of 250 μs with 60 μs magnetic-field rise time, we measured a 330 μs duration using a wire loop connected to a digital-to-analog acquisition board and sampled at 100 kHz (PCI-6036E, National Instruments, USA). This duration was confirmed using an oscilloscope.

## Results

To gain insight into the nature of any capacitor one would like to investigate its discharge dynamics. For the electrode-gel-skin interface, this can be observed by the decay of the induced artifact. This decay can be described by our artifact model and can be reconstructed by the fitting method proposed in the Methods. Here we provide evidence for the model by experimental tests for consistency and verification of the implications.

### Implication 1 and 2: Presentation of the power law decay on human skin and its statistical assessment

A fundamental ingredient of our model is the structure of human skin, which gives rise to the order-2 power law in the artifact decay.

A typical example of TMS-induced artifacts in EEG recordings from a human head is shown in Fig 4A and 4B, displaying traces of 32 EEG electrodes acquired at a sampling rate of 16 kHz. The artifacts appear in every trace, characterized by different shapes and amplitudes. The decay back to baseline can range from a few milliseconds to more than 100 ms. Some of the electrodes actually change the sign of the voltage during the decay. After averaging to reduce 50 Hz noise (Fig 4C) a power law behavior of order 2 becomes distinctly apparent in the decay (Fig 4D).

We proceed to examine TMS-EEG applied to the human knee. The results of the TMS-EEG application, overlaid by the fits, are shown in Fig 4E and 4G. Note first that the overall morphologies of the artifacts on the knee agree with those on the head (compare Fig 4A–4D, 4E and 4F). Visual inspection of the artifact-subtracted data shown in Fig 4G shows that the fitting procedure removes the large TMS artifacts, leaving almost flat lines. At the same time, it preserves, in all electrodes, small and slow drifts (e.g. electrodes 6, 27) as well as electrode-specific signals that may either be noise or could reflect physiological activity related to leg muscle tone (e.g. electrodes 15, 24).

For a quantitative assessment of the artifact-subtraction procedure, we tested the statistical significance of the fit. The significance of the fit was tested for times between *t*_1_ = 1.65 ms, corresponding to the earliest time point of artifact reconstruction, and a variable end time 2 ms < *t*_2_ < 100 ms. For each electrode trace *i* = 1, …, 28, we estimated the standard deviation *σ*_*i*_ from the samples acquired during the 100 ms period before the TMS pulse. We then tested the null hypothesis that the fit was correct for the time span *t*_1_ to *t*_2_, i.e. that the sum of squared fitting errors, normalized by $\sigma_{i}^{2}$, follows a *χ*^2^-distribution. The significance level *α* was set to 5%.

The results are presented in Fig 4H, which displays the longest times *t*_2,*max*_ up to which the test did not reject the null, i.e. accepted the fit. We see that each fit is accepted for at least 20 ms, and 12 out of 28 electrodes are accepted for the maximally tested time of 100 ms. For those electrodes that do not reach 100 ms, an increase occurs in the standard deviation of the artifact-subtracted data compared to that of the 100 ms pre-pulse time span for which the *σ*_*i*_’s are computed. For example, there seem to be drifts that are stronger than in that pre-pulse time span (e.g. electrodes 2, 21) or additional high-frequency noise (e.g. electrodes 10, 11) possibly related to TMS device recharging.

Finally, we compare the quality of our fitting method with that of [21], which fits each artifact by a sum of two decaying exponentials (this includes the possibility of a single exponential). This method also tests if a linear function will work, but this possibility is ruled out by the highly non-linear artifacts of Fig 4E. Fitting to a sum of two exponentials is shown in Fig 4I–4L. While the overall shape of the large artifacts is well captured (see Fig 4I), it is noticeable that the fits of the sum of exponentials decay to baseline much faster than the rational function fits of our model (compare Fig 4I, 4J to 4E and 4F).

Further analysis of the sum of two exponentials subtracted data in Fig 4K shows that in most electrodes there are spurious peaks, showing up immediately after the TMS pulse, within the first 20 ms (e.g. electrodes 8, 27). In a few electrodes a slow wave occurs, within 100 ms (e.g. electrodes 12, 22). Applying the statistical testing performed above (see Fig 4L) consequently reports rejection of 19 fits out of 28 for any time interval. Only 4 fits are accepted for 100 ms, of which 3 (electrodes 4, 24, 25) exhibit noise in the 100 ms before the pulse, which would increase the *σ*_*i*_ for these electrodes, thus reducing evidence against the null. 5 electrodes are significant for a time between 2 ms and 100 ms. Fig 4M shows the difference of the data reconstructed by our model and by fitting sums of two exponentials, demonstrating how fitting an exponential decay can introduce spurious artifacts.

Examination of the decay time constants we obtained for the fitting of two exponentials indicates that their values are not so different. This is related to an important issue in fitting a sum of two exponentials, which is the physical rationale for applying it. A behavior is typically described by a sum of two exponentials when two decay mechanisms dictate two disparate time scales that concatenate to provide a transition from one rapidly decaying exponential to a longer and slower second one. In fact, the ratio we measure of these two time scales is typically on the order of 4.8 (see Fig 4N), indicating a non-physical origin that determines the decay constants.

We therefore conclude that all discharge artifacts asymptotically turn into a power law of order 2. Even though the power law decay regime is eventually followed by an exponential decay regime (see the section on discharge regimes in the Methods), the latter does not contribute a significant part to the decay. This is in a clear disagreement with previous reports [3, 4, 17, 19–21].

### Implication 2 (continued): TMS artifacts from phantom heads (melons) deviate from the power law

Previous work compared human TMS-EEG artifacts to data acquired from a phantom head, using a muskmelon [26, 27] and a watermelon [28]. Following this practice, we recorded the artifacts using both a watermelon and a muskmelon (Fig 5A–5D). The general morphology of the artifacts in the melons is similar to that of human TMS artifacts in that there is an initial peak and decay. In fact, the initial one or two milliseconds shows the same shape, amplitude and timing. However, a substantial difference concerns the artifact decay.

As seen from a log-log plot, the artifact decay clearly deviates from a power law of order 1 or 2 (Fig 5A and 5B, dotted and dashed lines, respectively). The decay for the musk melon is faster than for the watermelon (about 30 ms and 250 ms, respectively), even though both decays shows a qualitative resemblance to human recordings (Fig 5C and 5D). We controlled for a possible influence of different conductivities of the inner tissue of the melon by repeating the experiments after fleshing out the watermelon. The results did not change either qualitatively or quantitatively.

The TMS device has a well-known recharging artifact (reported in [27]) that can be characterized by using these phantom head in terms of its appearance and morphology (Fig 5D). The recharging artifact appears at various intensities in around half of all our recorded epochs and consists of two biphasic ‘blips’ of up to 50 μV amplitude. The peak of the first blip varies within the first 10 to 20 ms after the pulse but the time interval between the blips is always exactly 20 ms.

We conclude that the initial peaks in melon artifacts are comparable to initial peaks in human EEG both in amplitude and morphology. Their decay, however, is strikingly different, and they do not display a power-law decay of order 2. This is all in excellent agreement with our model. Additionally, the timing and occurrence of the TMS device’s recharging artifacts can be characterized.

### Implication 3: TMS artifacts are affected by skin preparation (exfoliation or puncturing)

To check whether the power-law decay depends on specific dermal properties, we applied TMS before and after skin treatment. We reduced skin resistance and capacitance by delicate puncturing of the epidermis with a needle (see Methods). Two examples of trials that were performed on a single subject are shown in Fig 6A and 6B, and depict the artifact before (red) and after (blue) the skin puncturing. The trial envelopes (see Methods) in each condition are shown in Fig 6C and 6D. It is evident that the first half millisecond of the response with the pulse artifact is similar regardless of the skin treatment, but also that the decay artifact which follows is markedly different.

The amplitude of the decay artifact, starting 1.2 ms after the initial pulse, is generally decreased by the skin treatment. This is presented in Fig 6D, and is in agreement with [10]. We fit the artifact to both a power law and to an exponential, starting at the time when it first decayed below a fixed cut-off voltage *V*_*cutoff*_, and performing the fit for a fixed time span *t*_*fit*_. We apply this comparison to all electrode recordings in which the artifact amplitude is large enough, and does not change sign within the first 6 ms. This yields 26 and 20 of the 64 electrodes, depicted as dark traces in the two conditions in Fig 6E.

The values of *V*_*cutoff*_ and *t*_*fit*_ ensure that a large part of the decay is covered before the artifacts have reached the physiological signal range and that not too many electrodes are excluded. For Fig 6E and 6J, we chose *V*_*cutoff*_ = 1.5 mV (shown as dashed line in Fig 6E and 6J) and *t*_*fit*_ = 24.2 ms (corresponding to 200 samples). The analysis does not depend on these particular numbers. The particular power law that we fit has the form *a*/(*t* + *b*)^2^, were *a*, *b* are constants to be found by fitting. This form results from a simple approximation of the expansions (10) and (11) of the model description (see Methods).

To compare the power law versus the exponential fits for the decay, we consider their coefficient of determination *R*^2^ before and after skin punctuation, as shown in the bottom of Fig 6E. For comparison, we take the median of all *R*^2^’s before (power law: *R*^2^ = 0.999, exponential: *R*^2^ = 0.982) and after puncturing (power law: *R*^2^ = 0.997, exponential: *R*^2^ = 0.967). The power law has a better fit for both skin conditions, and since we are interested in the low amplitudes that are physiologically relevant, even small differences in *R*^2^ will be important.

We also investigated the effect of skin exfoliation on TMS artifacts, by rubbing the skin under the electrodes. This form of skin preparation is commonly applied to decrease electrode resistance and is a requirement for passive EEG systems. The effect of skin exfoliation is demonstrated in Fig 6F and 6G. The envelopes of the distribution of all electrode traces in each condition are shown in Fig 6H and 6I. In Fig 6J, we perform the same analysis as in the case of skin puncturing. A comparison of the median of all *R*^2^’s before (power law: *R*^2^ = 0.993, exponential: *R*^2^ = 0.985) and after exfoliation (power law: *R*^2^ = 0.994, exponential: *R*^2^ = 0.986) demonstrates again better quality of the power law fit.

We conclude that skin exfoliation leads to a significant scaling-down of artifact when compared to untreated skin, but it is not as effective as skin puncturing. Both skin preparation methods do not change the power-law decay.

### Implication 4: Placement of the reference electrodes shapes the power law

For the Biosemi and g.tec EEG systems used here, the voltage at a recording electrode is always subtracted by the reference electrode. Since both recording and reference electrodes can be affected by the TMS pulse, we tested whether there is a substantial alteration in the shape of the artifacts once the position of the reference electrode is changed. The stimulation site, the head position, and the locations of the recording electrodes were kept fixed. We stimulated at C2 and changed the location of the CMS and DRL electrodes while keeping their relative distance approximately constant. We recorded from the right hemisphere using 32 electrodes, with CMS and DRL first in their usual positions (see Methods and Fig 7A) and consecutively relocated to P3 and P7, to CP3 and TP7, to C3 and T7, to FC3 and FT7, to F3 and F7 as shown in Fig 7A.

The corresponding EEG traces recorded from electrodes F4 and C6 exhibit alterations in artifact shape on the order of several millivolts for different CMS-DRL positions (Fig 7B and 7C). The size of the artifact of electrode F4 gradually becomes smaller as CMS-DRL are moved more closely to F4 (Fig 7B). This is more complicated for electrode C6, where the artifacts even change direction (Fig 7C). Note that the artifacts induced by the conditions corresponding to the red and the pink lines in Fig 7C create similar artifact with opposite sign in C6.

Next, we looked into the dynamics of recording electrodes when the reference electrode is not affected by the TMS pulse. This was achieved by placing the reference electrode (CMS and its partner DRL, see Methods), including their respective electrode wires, far away from the TMS coil. We used 13 recording electrodes to cover the head, and the results are shown as red curves in Fig 7D–7F. The behavior is seen to be markedly different, the power law is now 1/*t* rather than 1/*t*^2^.

If we now compute the average of the electrode traces and subtract it from any one of them, then the power law of order 2 re-emerges, as shown by the green traces in Fig 7D–7F. In fact, this works with taking the difference between any two electrodes. Note that the green traces exhibit much less noise, resulting from the cancellation of the common mode signal by the subtraction. Furthermore, while the decay time of the green traces is comparable to the decay with the reference placed on the head, the decay time of the red traces is much longer, reflecting the fact that 1/*t* dominates 1/*t*^2^ for large *t*.

We conclude that the artifact shape is influenced by the relative position of recording and reference electrodes. Specifically, the decay in the artifact of a single recording electrode follows a power law of order 1 if the reference electrode is not affected by the TMS pulse, but if both are affected then the power law in the artifact is of order 2.

### Implication 5: Changing the sampling rate is compatible with the discharge artifact model

In the acquisition and processing of fast pulse and decay processes, we must consider the bandwidth and slew rate of the EEG amplifier. Fig 8 shows that varying the sampling rate leads to manifest differences in the initial peaks of the TMS artifacts. Specifically, at higher sampling rates they have larger amplitude, due to the increased bandwidth (Fig 8A). At 16 kHz, the artifact peaks reach the limits of the amplifier input range of ±262 mV. A concomitant spreading of these peaks (Fig 8A and 8B) appears, probably due to a slew-rate-related effect.

The general shape of the discharge artifacts is, however, not changed. This can be seen by shifting each artifact backwards by *t*_*align*_ until they coincide with the artifacts of 16 kHz (see Fig 8B–8D). We define |*D*_*i*_(*t*)| to be the (absolute values of) the difference between the signal from electrode *i*, measured at rate *S* and translated in time by *t* small steps of size 1000/16384 ms, and the signal of electrode *i* at rate 16kHz. We then minimize the sum of |*D*_*i*_(*t*)| over all electrodes, to find the alignment time *t*_*align*_(*S*). For the watermelon, *t*_*align*_(*S*) = 3.052, 1.099, 0.427, 0.122 ms for *S* = 1024, 2048, 4096, 8192 Hz, respectively. For the human head, *t*_*align*_(*S*) = 2.991, 1.160, 0.366, 0.122 ms, respectively. This was done on trial averages for both the watermelon and human head recordings for all acquisition rates.

We conclude that the sampling rate determines the precision and possible distortion of the recording of the actual artifact shapes, up to approximately 10 ms after the pulse for an acquisition rate of 1024 Hz compared to 16384 Hz. Two things are of note. First, shifts in time do not change the order-2 power law in the artifact decay tails, because the dominant contribution to a power law (*t* + *b*)^−2^ shifted by a constant *b* remains *t*^−2^. Second, our artifact removal algorithm is also stable with respect to time shifts in the artifacts. This is due to the fact that the class of functions with which the algorithm finds the optimal artifact fit consists of the linear span of suitable rational functions of type (1, 3). This class is invariant with respect to time shifts.

### Implication 6: Passive versus active EEG systems

We next demonstrate that the power-law decay of order 2 in the TMS artifacts can also be identified in data acquired by a passive EEG system. Specifically, we used a g.tec system that has annular ‘ring’ electrodes which are large, on the order of 15mm compared to the 2mm diameter of the Biosemi electrodes (see Methods). The EEG data were recorded from 9 electrodes at 38400 Hz (see Methods), and TMS stimulation was applied at three different sites, namely at C3, CPz, CP2 in 4, 3, 3 trials, respectively. Because the data had 50 Hz noise amplitudes of around 200 μV peak-to-peak, we emphasized the artifacts by fitting 50 Hz sine waves and subtracting them from the data.

Due to the high acquisition rate, the TMS pulse artifacts are well resolved (see Fig 9A). The g.tec artifacts show a qualitative resemblance to the Biosemi artifacts (compare Figs 9B and 9C to 4A). Importantly, also here the log-log plot is indicative of an order-2 power-law decay in the discharge artifacts as can be seen in Fig 9D. There is a large variation in the range of artifact durations, going from a few milliseconds for stimulation at CP2 (blue traces, Fig 9B) to several tens of milliseconds for stimulation at CPz (green, Fig 9C and 9D).

To verify that our model indeed applies to the artifact dynamics seen in the g.tec artifacts, we use our artifact removal method in the same way as for the Biosemi artifacts. A tricky point is that the simplifying assumption made previously in which the box function describing the electrode shape was approximated by a point, while valid for the Biosemi pin electrodes, is no longer justified for the large g.tec ring electrodes. The non-Gaussian charge distribution visibly manifests in the short-time artifact dynamics in the green traces in Fig 9A as marked by the arrow, but is in fact present in all g.tec artifacts to a varying degree.

While it is possible to adapt the method for non-point box functions, it requires careful derivation of the leading-order quantities for a ring-shaped box function modeling a ring electrode. To avoid a comprehensive theoretical analysis of the voltage difference for such a box function, we chose here a practical workaround, in which we rely on our model to define a time beyond which the decay is in its late stage and the power law decay is evident. We fit the data from a time *t*_0_ for each stimulation site, which we identify as the minimal time point beyond which the artifacts in all electrodes decay faster than a power law of order 1. In a log-log plot of the artifacts corresponding to a given stimulation site, this means that we look for a power law of order 1 (shown in magenta in Fig 9H) that is tangential to the slowest decaying artifact. These time points were identified visually for each stimulation site, as shown in Fig 9H and marked by vertical lines. The time point *t*_0_ was hence determined to be at 0.31 ms for CP2, 0.63 ms for C3, and 3.12 ms for CPz, respectively, following the end of the TMS pulse. Fig 9H shows the artifact fits for all electrodes of all trials starting from *t*_0_ of the corresponding stimulation site. Fig 9E, 9F and 9G each show the artifact fits for a single sample for a different stimulation site overlaid on the respective EEG data. Correspondingly, the reconstructed data after artifact subtraction are shown in Fig 9I, 9J and 9K.

To investigate the quality of the removal process, we again examine technical artifacts in the reconstructed data. In Fig 9C one can observe spiking artifacts likely originating from the TMS stimulator device, which occur with very similar timing. These artifacts can also be identified in the reconstructed data without distortion in Fig 9I, 9J and 9K (see also Fig 9E and 9F). Furthermore, a reconstructed biphasic muscle artifact can be observed in Fig 9J corresponding to stimulation site C3.

We conclude that our artifact model based on the skin capacitor can also be used for cleaning the data of the passive system with ring-electrodes. In particular, the model does not depend on a particular (active or passive) EEG system design. However, since the method relies on a simplifying assumption on the electrode geometry, for large ring electrodes the very short time behavior cannot be directly reconstructed. To apply our method, we thus needed to apply an ad-hoc procedure for selecting a minimal time point for applying the fit to the data.

### Implication 7: Separation of muscle and discharge artifacts

In Fig 10, we consider discharge artifacts when TMS was applied at more temporal head sites known to activate cranial muscles. Muscle activation also leads to large artifacts in EEG recordings, since muscle twitching results in mechanical motion of an electrode. The accompanying voltage change can exhibit drifts for over up to several tens of milliseconds and hence typically within the range of TMS-induced discharge artifact dynamics. We show here, using our artifact removal method, that there is a clear difference between the discharge artifacts and other muscle-activation-associated artifacts. This is used to determine the amplitudes and timing of muscle activation due to TMS stimulation.

Two subjects were tested, one at F1 and another at C5. In order to control for the performance of the reconstruction, we repeated each experiment also with strongly reduced artifacts, achieved by skin exfoliation and puncturing under each EEG electrode (see Methods). For unprepared skin, the artifacts shown as red lines in Fig 10A and 10E decay to the range of ±100 μV in all electrodes within 67 ms (for F1) and 85.4 ms (for C5). For treated skin, shown as blue lines in Fig 10A and 10E, this decay occurred within 5.2 ms (for F1) and 13.7 ms (for C5).

After artifact removal, we can describe the muscle artifacts, appearing as large biphasic waveforms. The duration of the biphasic muscle artifacts occurs within 7 ms and 13 ms for stimulation at F1 and C5. Most importantly, the amplitudes of muscle artifacts are about 1.2 mV for stimulation at F1 (in Fig 10C and 10D) and 4 mV stimulating at C5 (Fig 10G and 10H). This is orders of magnitude below the maximal voltage of the TMS artifact, indicating that muscle artifacts are dominated by the discharge ones.

We conclude that the largest contribution (by more than an order of magnitude) following the TMS pulse results from skin capacitor relaxation. The corresponding discharge artifact can be clearly distinguished from any artifacts associated with cranial muscle activation, which additively contribute to the discharge artifacts.

Since we effectively remove the discharge artifact, we are able to discern also the decay process of the muscle artifacts. Specifically, the lines decaying immediately following the biphasic muscle artifact, starting at around 13 ms after TMS at C5 (see Fig 10G) are not part of the discharge artifacts (compare artifacts in Fig 10G and 10H to the fits in Fig 10F). The reconstructed data for treated and untreated skin exhibit the same shape for the muscle artifact, up to small differences as shown in Fig 10C, 10D, 10G and 10H.

### Consistency test 1: Reconstruction of technical artifacts

To test the performance and quality of the method, we utilize the presence of known, precisely timed technical artifacts with sizes of physiological brain activity which are ‘riding’ on the discharge artifacts. The original data after removal of the average is shown in linear and log-log plots in Fig 11A and 11B, while the data after artifact removal according to our method is presented in Fig 11C–11F for a variety of time windows and averaging conditions. The reconstructed technical artifacts related to the TMS power supply recharging ‘blips’ reported in [27] and the power-line 50 Hz are apparent in Fig 11C and 11D, respectively. The ‘blips’ can clearly be identified in Fig 11C. Five electrodes affected by 50 Hz noise were reconstructed in Fig 11D without visible distortion. In Fig 11F we show the average over 99 trials, confirming and portraying the effect that the TMS application had on brain EEG. All trials used for averaging were first cleaned from the discharge artifacts with our method, and afterwards band-pass filtered between 1 and 300 Hz and notch-filtered at 50 Hz.

### Consistency test 2: Reconstruction of TMS-evoked potentials

Fig 12 presents EEG data from TMS applied at the motor cortex (see Methods). Shown in Fig 12A are artifact fits (colored lines) to average-subtracted but otherwise raw EEG data of a single trial (gray) after TMS application. Artifacts were fitted over the time span between 1.6 ms to 20 ms. The artifact decay can last for tens of milliseconds, shown in Fig 12B and in the corresponding log-log plot in Fig 12C. The reconstructed data of the single trial obtained from artifact fit subtraction are shown in Fig 12D–12F, at different time windows.

The effect of cranial muscle activation is apparent in the large negative deflections to below -70 μV between 6 and 11 ms that is observed in electrodes F4, F6, F8 (see Fig 12D). To verify that these are indeed the previously reported TMS-evoked potentials (TEPs) associated with motor cortex stimulation, we interpolated the artifact-subtracted data during the first 1.6 ms following the TMS pulse and afterwards band-pass and notch-filtered from 1 to 300 Hz and at 50 Hz, respectively. The outcome is shown in Fig 12E and 12F for both the filtered single trial and the average over 20 filtered trials. TEPs reported in [4] to be N15, P30, N45, P60, N100, and P180 can be identified.

## Discussion

In this paper, we present a quantitative physical model of the TMS artifacts in EEG. This model describes the artifacts as discharge voltages of capacitances that were charged by the TMS pulse. The structures within the skin are the major sources of this capacitance. The paper provides a detailed description of the model and implications that arise from the model. These are then tested with real data, demonstrating that the model can reproduce experimental results. Our model can also be used to simulate discharge artifacts (Fig 3), and furthermore provides analytic formulas for the power laws. To utilize the model to derive an artifact reconstruction method, we use approximations and simplifications of the formulas of the model. This method achieves artifact removal by first fitting analytic expressions to the artifacts and then removing them from the data by subtraction.

To summarize, we have presented seven implications that arise from the model, as well as two consistency checks, and have verified all these.

We first demonstrated that artifacts reconstructed by our model pass a statistical test for TMS-EEG artifacts measured on a human knee (Fig 4). This gives evidence to the idea that their origin is discharge of skin capacitances, and demonstrates that the dominant decay dynamics of the discharge voltage follows a characteristic power law, while the terminal exponential decay regime does not play a relevant role. Second, surfaces with non-skin structure do not exhibit the same power law (Fig 5). Third, we showed that exfoliation and puncturing of the skin do not affect this power law, although they have an effect on the artifact shapes (Fig 6). Fourth, we showed that the it is the subtraction of the voltage of the reference electrode that causes the power law to be of order 2 (Fig 7). For the fifth and sixth implications, we demonstrated that the sampling rate and amplifier design do not change the power law (Figs 8 and 9). The seventh implication was that muscle artifacts are distinct and can be separated from the discharge artifacts (Fig 10).

The first consistency check was a faithful reconstruction of technical artifact (Fig 11), including the 50 Hz noise from the power line and the two bi-phasic recharging blips of the TMS power supply, which are always separated by 20 milliseconds. The second was the ability to measure TMS-evoked potentials, as previously reported in the literature (Fig 12).

### Origin of the power law

The two-dimensional spatial extent of the skin interface is the main physical feature of our model. While the view of skin as a resistive-capacitive layer is well-established, it is commonly modeled by a single leaky capacitor (see [6]). The decay of a single leaky capacitor is, however, exponential and thus cannot reproduce the power law. In contrast, a large enough configuration of many resistor and capacitance elements may approximate it for intermediate time scales. Indeed, the electrical properties of additional layers of the tissue underneath the epidermis and between the electrodes may be described by a network of resistors and capacitors [33]. The success of our model in reproducing the TMS-induced artifacts as the voltage evolution of a resistive-capacitive layer lies in approximating the simplistic skin model (Fig 1A) by a periodic network of circuit elements. The voltage in networks of this type and their homogenization limits are described by the class of transmission line models and cable equations in one and higher dimensions [25]. The voltage in our model, a two-dimensional cable equation, exhibits a power law decay regime. While the presence of resistors in the resistive-capacitive layer eventually leads to an exponential decay regime, our data demonstrate that this regime does not contribute a significant part to the decay.

Indeed, a non-exponential voltage decay was observed long ago in the human wrist [34], following a square wave current injection of a few milliseconds duration by two stimulation electrodes. This voltage lasted much longer than the actual current pulse. This result was demonstrated to hold for square current and voltage pulses of 200 μs duration [35]. However, a power-law in the decay of the discharge voltage of the skin capacitor after direct skin stimulation was, to the best of our knowledge, not reported before. Indeed, the full artifact is only visible using a potentiometer with a detection range of hundreds of millivolts and a resolution of at most a few microvolts. Therefore, a power law behavior may not have been noted. The use of an exponential function to fit a power law may lead to small time constants and result in a strong underestimation of the actual decay duration. For example, as can be seen in the log-log plots of Figs 11 and 12, the initial decrease of the artifacts is at least one decade within the first 10 ms. If the decay were taken as exponential and only the first decade considered, its corresponding time constant would be estimated to be 5 ms or less.

The finding of a power-law decay as opposed to exponential decay has profound consequences, and is important. Exponential decay, such as the weighted sum of several exponential functions, occurs within a specified time scale, and will therefore at some point decrease much faster than power-law decay. In fact, it will decay much faster than the inverse of any power law can grow. As a consequence, any model that assumes exponential decay cannot remove the discharge artifacts correctly and hence typically introduces additional artifacts in the data.

### Skin preparation by puncturing and exfoliation

In the literature, different effects of skin preparation have been reported on the pulse artifact. Here we showed that the very fast initial pulse artifact is not changed in amplitude following skin puncture (Fig 6A and 6C), pointing at an effect unrelated to skin. This complements the report of [10], where the actual amplitude of the pulse artifact could not have been detected because a ‘sample-and-hold’ system was used.

In contrast, we showed that the discharge artifact is downscaled but that it still decays with a power law (Fig 6E). Because a power law has no time constant, the artifact length is not decreased, rather the time for the artifact to reach the range of physiological EEG activity is reduced, confirming [10]. The puncture reduces the skin capacitance, so that less charge accumulates in the skin from the pulse, resulting in lower initial amplitude of the artifact dynamics. This causes downscaling of the discharge artifact.

Another skin preparation that modifies the resistive-capacitive properties of skin is exfoliation (‘rubbing’). As in the case of puncturing, we find that the initial pulse artifact is not changed (Fig 6F and 6H). The discharge artifact (Fig 6G and 6I) is downscaled like for puncturing, but the effect is not as strong (compare Fig 6D and 6I). Importantly, skin exfoliation also does not change the power law decay (Fig 6J).

### TMS artifacts in active versus passive EEG systems

We showed that both an active (Biosemi) and a passive (g.tec) EEG system display similar discharge artifact decays (see Methods). It is unclear why the g.tec artifacts exhibit a shorter duration (compare Figs 11B and 9B). One possibility is that differences in amplifier electronics and how they contend with the very large amplitudes induced by TMS cause this difference, but we do not think this is the correct answer.

Rather, we believe the skin preparation, which is practically always required with passive systems, is the origin of this difference. Indeed, an active EEG system with skin preparation as in Fig 6 demonstrates that exfoliation reduces the discharge artifact durations also in an active system, with measured values similar to those observed in the passive system (Fig 9). Most important, a power law in the discharge artifacts can be clearly identified also for the passive system. We therefore propose that the physical mechanisms underlying the discharge artifacts recorded with the active as well as the passive system are the same.

### The relationship between cranial muscle activation and discharge artifacts

In the TMS literature, there is a controversy [5, 11] regarding the relative contributions of the different artifact components to the overall TMS-induced artifacts in EEG. The question is whether the dominant contribution to the long decay in the artifact is mainly due to the electrode-gel-skin interface [10], to muscle activation [5, 14, 28], or to electrode motion following muscle activation [11].

This controversy is difficult to resolve, but our approach enables us to separate artifact components that are caused by muscle activation from those that are due to skin capacitor discharge. This makes it possible to compare their respective characteristic amplitudes and shapes. Fig 10 shows quite clearly that the large-amplitude part of the artifact results from the skin capacitor and is orders of magnitudes larger than the biphasic muscle artifact. The discharge artifact amplitudes can reach up to a few hundreds of millivolts for the sampling rates and electrodes used here (Figs 4F and 9A). In contrast, the amplitudes of the biphasic muscle artifacts reach between several hundred microvolts (Figs 12D and 9J) to a few millivolts (Fig 10), as recorded here.

Achieving this kind of disambiguation between discharge and muscle artifacts by eye, without a model, may not always be possible. Ambiguities often arise from the fact that the amplitudes of the discharge artifacts can come in a variety of shapes and form, spanning from a few tens of microvolts to a few hundreds of millivolts (Fig 12A). They may also have a roughly biphasic appearance (e.g. Fig 12A), or appear to be smaller than they actually are due to sampling rate (Fig 4F). Similarly, muscle artifacts can resemble the discharge artifacts since their duration also spans the range of around 10 ms (Figs 12F and 9J) to several tens of milliseconds (Fig 10G and 10H). Indeed, the decay to baseline between 13 ms and 35 ms of the muscle artifacts in Fig 10G and 10H most likely results from mechanical motion of the electrodes [6] after muscle twitching [11]. This makes it difficult to visually determine the origin of an artifact component without a physical model for the discharge artifacts.

### Variations in the initial conditions of the model

The initial voltage distribution at each electrode is created because the magnetic pulse charges the resistive-capacitive layer at the skin-electrode interface. The initial conditions of the mathematical problem are the actual voltage distribution, which in turn depends on a number of factors. The potential of the electrodes is initially determined by current induction in the metal of wires and electrodes during the magnetic pulse. This leads to an electron density inside the metallic electrode surface which is governed by the field line density of the electric field. Ions then accumulate at the opposing gel interface of the electrode. Because the scalp surface is parallel and near the bottom face of the metal cylinder, its potential dominates also the formation of a rotationally symmetric charge density on the capacitive layer of the epidermis.

Once the magnetic pulse is terminated the resulting charge density determines the initial voltage distribution that we use for our model. For simplicity, we approximate the initial voltage by a Gaussian distribution. Within this picture the barycenter of this Gaussian is aligned with the longitudinal axis of the metal cylinder. However, in practice deviations from this picture may occur due to skin anisotropy or an external electric field, which may shift this barycenter or distort the initial distribution. We address these contributions by allowing for possible displacements of the barycenter of the Gaussian voltage distribution away from the longitudinal axis of the cylindrical electrode.

### Limitations and advantages of the method and future research

A present limitation of our artifact-removal method that we derived from the physical model is that we use a rather coarse approximation of the model to fit the artifacts. In particular, the electrode shape was approximated by a point even though our artifact model can generally treat electrodes of any shape (using a box function). Generally this is acceptable, since diffusion ensures that, with time, any initial charge distribution located inside a confined area effectively becomes a Gaussian. Nevertheless, for short times, the deviation from Gaussian shape may be significant. Correspondingly, the initial time that must be skipped (currently around 1.6 ms at 8 kHz sampling rate with pin-type Biosemi electrodes) may vary. Our method can be improved by deriving a more precise short-time expansion of the integral in Eq (7) than is done in the section on our artifact removal method (see Methods). Furthermore, despite the robustness of the choice of the time window in which fitting is performed, the development of statistical estimators to find the optimal fitting window is desirable.

The fitting method has two main strengths. The first is conceptual in that the method is based on a quantitative physical model rather than on statistical assumptions about the data and the artifacts (as is done in ICA). Specifically, this adds reliability to the fit performance when the long power-law artifact tails enter the physiological range for EEG of ±50 μV. In this range, the artifact can even continue for more than 150 ms (see e.g. Fig 12B) before it is less than ±2 μV. Our method reliably determines the complete artifact tail. This differs from other artifact reconstruction methods that require human intervention to make a decision on the duration or size of the artifact in the physiological range of EEG data.

The second strength lies in its ability to accommodate a large degree of automatized deployment. The fitting procedure can be applied to raw data, and will typically tolerate many other EEG artifacts such as eye blinks, 50 Hz noise or drifts, as long as they are much smaller than the TMS-induced discharge artifacts. The only parameters to be determined are the time range for fitting of each signal. The method is rather robust to varying the fitting ranges, so that a single range can usually be determined for each electrode by using a single trial. Fitting is then applied automatically to all subsequent trials using the same fit parameters.

While our model describes the skin capacitor discharge dynamics, it lacks a description of the physical processes providing the exact initial voltage distribution at each electrode for given wire locations, coil geometry, coil position, and magnetic stimulation intensity. Rather, the initial conditions are assumed to be given in the form of parametrized Gaussians convolved with a box function. It remains to be verified that this assumption is not too restrictive in that the initial conditions enforced by the TMS pulse can always be approximated in this form. To describe different charge distributions underneath each electrode, different box functions may be used for different electrodes. They should be normalized by their integral to assure the mutual cancellation of the first-order power-law contribution. Furthermore, the model does not include the possible existence of subcutaneous electric fields. Experimental evidence from current pulse injection in the human wrist [34, 35] demonstrates that the discharge voltage of the skin capacitor following this injection can be detected by recording electrodes placed centimeters away from the stimulation site. This was explained in [35] by large voltage gradients in the deep layers of the skin. It is therefore also a possibility that the discharge voltage of the skin capacitor after a TMS pulse at one EEG electrode can also affect neighboring electrodes. This potential effect is not included in our model.
